# Cyclodepsipeptides and Sesquiterpenes from Marine-Derived Fungus *Trichothecium roseum* and Their Biological Functions

**DOI:** 10.3390/md16120519

**Published:** 2018-12-19

**Authors:** Yuan-Ming Zhou, Guang-Lin Ju, Lin Xiao, Xiang-Fei Zhang, Feng-Yu Du

**Affiliations:** 1 College of Chemistry and Pharmacy, Qingdao Agricultural University, Qingdao 266109, China; zym7410@163.com (Y.-M.Z.); jgl2018666@163.com (G.-L.J.); xiaolin_qd@163.com (L.X.); 88121029@163.com (X.-F.Z.); 2 Analytical and Testing Center, Qingdao Agricultural University, Qingdao 266109, China; 3 Shandong Province Key Laboratory of Applied Mycology, Qingdao Agricultural University, Qingdao 266109, China

**Keywords:** marine-derived fungus, cyclodepsipeptides, sesquiterpenes, cytotoxic activity, nematicidal activity

## Abstract

On the basis of the ‘one strain, many compounds’ (OSMAC) strategy, chemical investigation of the marine-derived fungus *Trichothecium roseum* resulted in the isolation of trichomide cyclodepsipeptides (compounds 1–4) from PDB medium, and destruxin cyclodepsipeptides (compounds 5–7) and cyclonerodiol sesquiterpenes (compounds 8–10) from rice medium. The structures and absolute configurations of novel (compounds 1, 8, and 9) and known compounds were elucidated by extensive spectroscopic analyses, X-ray crystallographic analysis, and ECD calculations. All isolated compounds were evaluated for cytotoxic, nematicidal, and antifungal activities, as well as brine shrimp lethality. The novel compound 1 exhibited significant cytotoxic activities against the human cancer cell lines MCF-7, SW480, and HL-60, with IC_50_ values of 0.079, 0.107, and 0.149 μM, respectively. In addition, it also showed significant brine shrimp lethality, with an LD_50_ value of 0.48 μM, and moderate nematicidal activity against *Heterodera avenae*, with an LC_50_ value of 94.9 μg/mL. This study constitutes the first report on the cytotoxic and nematicidal potential of trichomide cyclodepsipeptides.

## 1. Introduction

In the search for novel bioactive metabolites from various natural resources, marine-derived fungi have gained increasing attention due to their potential capacity to produce structurally unique and biologically active metabolites [1,2]. However, metabolites isolated from marine-derived fungi generally perform far below their biosynthetic potential, indicating the existence of silent biosynthetic pathways [3,4]. The culture strategy of ‘one strain, many compounds’ (OSMAC) is able to significantly increase the chemical diversity of fungi [5,6,7]. One successful application was the isolation of a series of cytotoxic cytochalasins from a marine-derived fungus *Spicaria elegans* KLA03, via the modification of the culture media and the addition of tryptophan [8,9,10].

**Figure 1:**
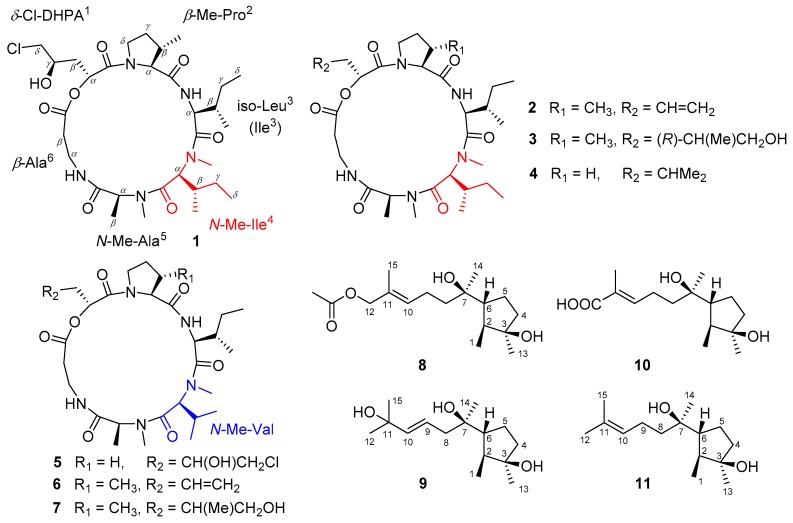
Compounds 1–4 from the liquid PDB medium, 5–10 from the solid rice medium, and the reference Compound 11 (cyclonerodiol).

Cyclodepsipeptides of the trichomide and destruxin classes are all cyclic hexadepsipeptides containing an α-hydroxy acid, a β-alanine, and four α-amino acids [11,12,13]. The characteristic structure difference between the two classes is the replacement of the *N*-methyl-isoleucine residue in trichomides with a *N*-methyl-valine in destruxins [11,12]. In 2013, Tan and his co-workers reported the isolation of trichomides A–C with significant immunosuppressive activities [11], while destruxins exhibited various biological activities, including V-ATPase inhibition [13], antiviral [14], phytotoxic [15], and insecticidal activities [16]. Cyclonerodiol sesquiterpenes, generally isolated from the genera *Trichoderma* and *Trichothecium*, have been reported to exhibit anti-inflammatory, antiallergic, and antimicrobial activities [17,18,19]. During the ongoing search for bioactive leading compounds [20,21,22], the PDB (Potato-Dextrose Broth) medium extract of a marine-derived fungus *Trichothecium roseum* attracted our attention because of its cytotoxic and nematicidal potential. From this extract, bioactive cyclodepsipeptides of the trichomide class (compounds 1–4) were isolated (Figure 1). Based on the ‘one strain, many compounds’ (OSMAC) strategy, a different rice medium extract showed a different metabolite profile and further structural types, including cyclodepsipeptides of the destruxin class (compounds 5–7) and cyclonerodiol sesquiterpenes (compounds 8–10) (Figure 1). The isolation, structural elucidation, and biological evaluation of the isolated compounds (compounds 1–10) are discussed herein.

## 2. Results and Discussion

### 2.1. Structural Elucidation

The molecular formula of trichomide D (compound 1), C_31_H_52_N_5_O_8_Cl, was obtained using HRESIMS (Figure S2 in the Supplementary Materials). The one-dimensional NMR data (Table 1) exhibited six carbonyl carbons (*δ*_C_ 173.6, 173.6, 172.4, 172.1, 170.8, 170.4) and two amide *NH* protons (*δ*_H_ 8.56, 7.03), indicating the presence of a cyclic hexadepsipeptide skeleton.

**Figure 2:**
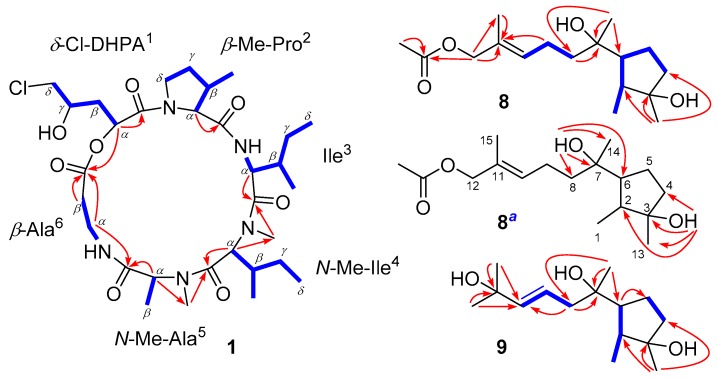
The key ^1^H–^1^H COSY (blue bond lines) and HMBC (red arrows) correlations of 1, 8, and 9. *^a^* measured in DMSO-*d*6.

**Figure 3:**
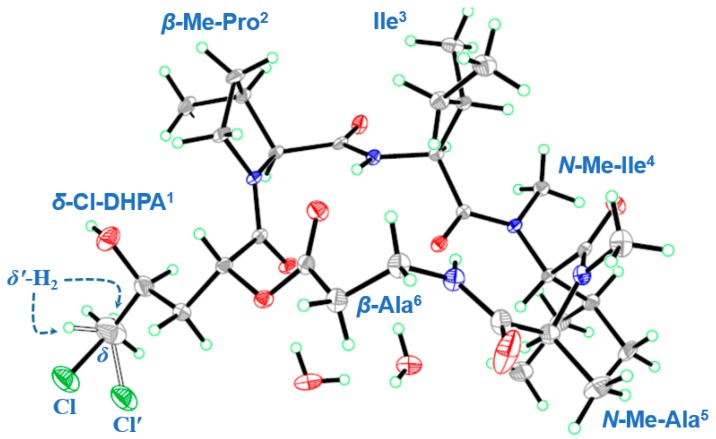
X-ray crystal structure of compound 1.

Further detailed analyses of one-dimensional and two-dimensional NMR data (Figures S3–S7) resulted in the identification of one *δ*-chloro-*α*,*γ*-dihydroxypentanoic acid (*δ*-Cl-DHPA^1^) and five amino acids, including *β*-Me-proline (*β*-Me-Pro^2^), isoleucine (Ile^3^), *N*-Me-isoleucine (*N*-Me-Ile^4^), *N*-Me-alanine (*N*-Me-Ala^5^), and *β*-alanine (*β*-Ala^6^) residues (Figure 2). Based on the characteristic ^13^C NMR signal of *δ*_C_ 48.3, the chlorine atom should be connected to the *δ*-CH_2_ group in the DHPA^1^ residue. The above-mentioned residues were preliminarily connected by the observed HMBC cross-peaks from *α*-CH or *N*-Me signals to the carbonyl carbons, indicating the presence of a cyclic hexadepsipeptide with the sequence of cyclo-(*δ*-Cl-DHPA^1^-*β*-MePro^2^-Ile^3^-*N*MeIle^4^-*N*MeAla^5^-*β*-Ala^6^). The structure of compound 1 was unambiguously confirmed by single-crystal X-ray diffraction using Cu Kα radiation, which showed *R* configurations of both *α*-CH and *γ*-*OH* in the *δ*-Cl-DHPA^1^ residue (Figure 3). The amino acid units in compound 1 were all assigned as *L*-configured (Figure 3).

Cyclonerodiol C (compound 8) was confirmed to have the molecular formula C_17_H_30_O_4_ by its HRESIMS data (Figure S8), implying three degrees of unsaturation. The one-dimensional NMR (Figures S9 and S10) and HSQC spectra (Figure S12) showed the characteristic signals of one carbonyl (CO, *δ*_C_ 171.5) and two olefinic carbons (CH, *δ*_C_ 129.6/*δ*_H_ 5.47 and C, *δ*_C_ 129.9) (Table 2), which were responsible for two degrees of unsaturation. Therefore, a remaining degree of unsaturation due to one carbon ring can be deduced. The consecutive COSY cross-peaks (Figure S11) from H-1 to H-2, 6, 5, and 4, as well as the HMBC peaks (Figure S13) from H-13 to C-2, 3, and 4, could be identified as one cyclopentane residue (Figure 2). Detailed analyses of the one- and two-dimensional NMR data (Figures S9–S13) suggested that the structure of compound 8 was similar to that of cyclonerodiol (compound 11), except that the signals of the methyl group (*δ*_C_ 26.1/*δ*_H_ 1.67) in cyclonerodiol (compound 11) were absent from the ^1^H and ^13^C NMR spectra of compound 8 [23]. Instead, additional oxymethylene (CH_2_, *δ*_C_ 69.9/*δ*_H_ 4.45) and acetoxyl groups (CH_3_, *δ*_C_ 19.4/*δ*_H_ 2.08 and CO, *δ*_C_ 171.5) were observed in the spectrum of compound 8 (Table 2). The key HMBC correlation between H-12 and the carbonyl carbon further confirmed the linkage between C-12 and the acetoxyl group (Figure 2). The NMR data of compound 8 (Figures S14–S17) were also measured in DMSO-*d*6, showing two *OH* signals of *δ*_H_ 3.81 and 3.89 (Table S1). The connections between 3-OH (*δ*_H_ 3.81) and C-3, as well as between 7-OH (*δ*_H_ 3.89) and C-7, were confirmed by the HMBC cross-peaks from 3-OH to C-3, 4, and 13, as well as from 7-OH to C-7, 8, and 14, respectively (Figure 2).

The molecular formula of compound 9, C_15_H_28_O_3_, was determined via HRESIMS (Figure S19). The one-dimensional NMR (Figures S20 and S21) and HSQC (Figure S23) data exhibited marked similarities to those of cyclonerodiol (compound 11) [23], except for the chemical shifts of the two olefinic carbons (CH-9, *δ*_C_ 122.1/*δ*_H_ 5.70 and CH-10, *δ*_C_ 142.3/*δ*_H_ 5.70), and the presence of another oxygenated quaternary carbon (C-11, *δ*_C_ 70.8) in compound 9 (Table 2). CH-9 in the double bond was connected to CH_2_-8, based on the COSY correlation (Figure S22) between H-8 and H-9, while CH-10 was linked to the quaternary C-11 by the key HMBC cross-peaks (Figure S24) from H-12 and 15 to C-10 and C-11. Thus, the planar structure of compound 9 was confirmed, and it was named cyclonerodiol D.

**Figure 4:**
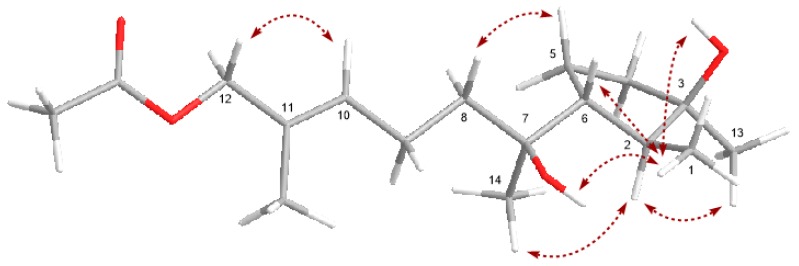
Key NOE correlations of compound 8.

**Figure 5:**
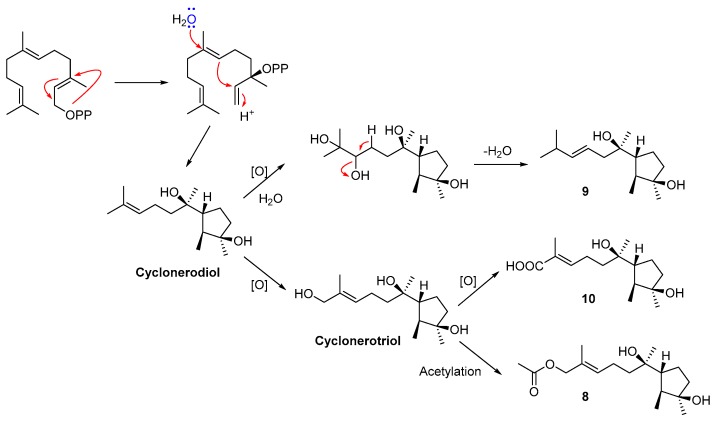
Plausible biosynthetic pathways of cyclonerodiol sesquiterpenes, compounds 8–10.

The relative configuration of the cyclopentane residue in compound 8 was deduced by the NOESY experiment (Figure 4 and Figure S18). The key NOE correlation between H_3_-13 and H-2 suggested an *α* orientation of these protons, while the cross-peaks from H_3_-1 to H-6 and 3-OH indicated a *β* orientation. The *E*-geometry for the double bond in compound 8 was also confirmed by the NOE correlation between H-10 and H_2_-12. The relative configuration of C-7 in compound 8 was initially assigned via the NOE cross-peaks from H_3_-14 to H-2, from 7-OH to H_3_-1, and from H_2_-8 to H_2_-5 (Figure 4, Figure S18), which suggested that the rotation of the single bond C6−C7 was restricted by the surrounding groups [24]. Although the specific rotation of compound 8 was similar to that of cyclonerodiol (compound 11) (${\left[\alpha \right]}_{D}^{24}$ = −28.3, *c* 0.86 for 8 *versus* [${\left[\alpha \right]}_{D}^{24}$ = −21.0, *c* 1.04 for cyclonerodiol, both determined in CHCl_3_) [23], evidence for the absolute configuration of compound 8 was still weak. However, the biosynthetic pathway of cyclonerodiol (compound 11) in *T. roseum* had been already confirmed [25,26,27], suggesting that the cyclonerodiol sesquiterpene compound 8 could also been biosynthesized via the same pathway in *T. roseum*. Therefore, the plausible biosynthetic pathway of compound 8 was deduced from cyclonerodiol (compound 11), further indicating the same absolute configurations between compounds 8 and 11 (Figure 5). In addition, comparison of the NMR data between compound 8 and cyclonerodiol (compound 11) showed marked similarities. Based on the above-mentioned analyses, the absolute configuration of compound 8 is likely the same as cyclonerodiol (compound 11). On the basis of the same analyses of NOE correlations and the plausible biosynthetic pathway of compound 9 (Figure 5), its absolute configuration is likely also the same as that of compound 8 and cyclonerodiol (compound 11). Although the cotton effects (CEs) from 200 nm to 240 nm in the measured CD spectrum of compound 9 were relatively low, its CEs (negative CE near 210 nm, and positive CE near 230 nm) were very similar to those of the calculated CD spectrum. The result of this ECD (Electronic Circular Dichroism) calculation, more or less confirms the absolute configuration of compound 9 as 2*S*, 3*R*, 6*R*, 7*R*, and 9*E* (Figure S25). Detailed methods of the ECD calculation are also described in the Supplementary Materials.

Besides the above-mentioned three novel compounds (compounds 1, 8, and 9), three known trichomide cyclodepsipeptides were isolated from the PDB medium: destruxin A5 (compound 2) [11], trichomide A (compound 3) [11], and homodestruxin B (compound 4) [11]. Three typical destruxin cyclodepsipeptides, destruxin chlorohydrin (compound 5) [28], roseotoxin B (compound 6) [29], and C (compound 7) [30], and one cyclonerodiol sesquiterpene, ascotrichic acid (compound 10) [31] were isolated from the rice medium. The structures of these compounds were determined by detailed analyses of their spectroscopic data and comparisons with previously published reports.

### 2.2. Biological Evaluation

The novel compounds (compounds 1, 8, and 9) were evaluated for their cytotoxic activities against five human cancer cell lines (MCF-7, SW480, HL-60, A-549, and SMMC-7721) [32,33,34]. Compound 1 showed significant cytotoxicity against MCF-7, SW480, and HL-60, with IC_50_ values of 0.079, 0.107, and 0.149 μM, respectively—better than the positive control of cisplatin (Table 3). However, compounds 8 and 9 were inactive in the cytotoxic assay (IC_50_ > 40 μM). This is the first report on the cytotoxic activity of trichomide cyclodepsipeptides. Although the cytotoxic mechanisms of trichomides have not yet been revealed, the cytotoxic mechanisms of the structurally similar destruxins have been reported to be associated with the inhibition of the phosphoinositide-3-kinase (PI3K)/Akt pathway, and the disturbance of the intracellular redox balance. Therefore, trichomide cyclodepsipeptides might show the similar cytotoxic mechanisms to destruxins [13,35].

Brine shrimp (*Artemia salina*), an aquatic species characterized by high sensibility to toxins, can be used as a model organism for quick preliminary insecticidal screening [36,37]. Therefore, in order to identify the leading insecticidal compounds, all of the isolated compounds (compounds 1–10) were evaluated for lethal activity against brine shrimp, and furthermore, for nematicidal activity against *Heterodera avenae* [38] (Table 4). In the brine shrimp assay, the cyclodepsipeptide compounds 1, 2, and 4–6 exhibited significant lethal activity, with LD_50_ values of 0.48, 0.74, 3.22, 2.47, and 2.81 μM, respectively. The nematicidal assay showed that compounds 1 and 2 exhibited moderate activity, with LC_50_ values of 94.9 and 143.6 μg/mL, respectively. Compounds 1 and 2 exhibited obviously better insecticidal and nematicidal activity against brine shrimp and *H. avenae* than the other cyclodepsipeptides, which was probably due to the structural diversity of the DHPA^1^ residues and the presence of *N*-Me-Ile^4^ residue in the trichomide cyclodepsipeptides.

However, only compound 9 showed moderate antifungal activity against *Valsa mali*, with an MIC value of 64 μg/mL. The trichomides (compounds 1 and 4) and sesquiterpene (compound 8) exhibited weak bioactivities against *V. mali* and *Rhizoctonia cerealis*, with MIC values from 128 to 256 μg/mL (Table S2). None of the isolated compounds exhibited activity against *Fusarium. oxysporum* f. sp. *vasinfectum*.

## 3. Experimental Section

### 3.1. General Procedures

One- and two-dimensional NMR spectra were recorded at 500 MHz and 125 MHz for ^1^H and ^13^C respectively, using a Bruker Avance III spectrometer (Bruker Biospin Group, Karlsruhe, Germany) with TMS as internal standard. HRESIMS were determined using a Bruker impact II ESI–QTOF mass spectrometer (Bruker Daltonik, Bremen, Germany). Optical rotations were obtained using a Jasco P-1020 digital polarimeter (Jasco Corporation, hachioji-shi, Tokyo, Japan). ECD spectra were acquired using a Chirascan spectropolarimeter (Applied Photophysics Ltd., Surrey, UK). Column chromatography (CC) was performed with Si gel (200–300 mesh; Qingdao Haiyang Chemical Co., Qingdao, Shandong, China), Lobar LiChroprep RP-18 (40–63 μm; Merck, Kenilworth, NJ, USA), and Sephadex LH–20 (18–110 μm; Merck, Kenilworth, NJ, USA). Semi-preparative HPLC was performed using a Dionex HPLC system equipped with a P680 pump, an ASI-100 automated sample injector, and a UVD340U multiple wavelength detector controlled using Chromeleon software, version 6.80 (Dionex Corporation, Sunnyvale, CA, USA).

### 3.2. Fungal Material

The fungal strain *T. roseum* was isolated from marine driftwood collected from the intertidal zone of Lingshan Island, Qingdao, China in November 2013. The fungus was identified on the basis of morphological characteristics and molecular analyses of ITS [20]. The strain was preserved in the Natural Products Laboratory, College of Chemistry and Pharmacy, Qingdao Agricultural University.

### 3.3. Fermentation and HPLC Analyses

Fresh mycelia of the fungus were statically fermented at 28 °C for 30 days on the liquid PDB and solid rice media. The liquid culture was conducted in 40 × 1 L conical flasks containing 300 mL of PDB medium (1000 mL natural seawater, 20 g glucose, and 200 mL potato juice, pH 6.5−7.0), while the solid one was kept in 40 × 1 L flasks containing rice (100 g/flask), peptone (0.6 g/flask), and natural seawater (100 mL/flask).

Ethyl acetate (EtOAc) extracts of the two fermentations were analyzed using HPLC with a MeOH-H_2_O eluting gradient and a detection wavelength of 225 nm (Figure S1). Details are described in the Supplementary Materials.

### 3.4. Extraction and Isolation

The PDB culture was exhaustively extracted using EtOAc to obtain a crude extract, which was fractionated via silica gel vacuum liquid chromatography (VLC) with a chloroform/MeOH gradient (100:1, 50:1, 20:1, and 10:1) to yield four fractions (Frs. 1–4). Fr. 3 was purified via CC over RP-C18, eluting with a MeOH-H_2_O gradient (from 2:8 to 1:0) to obtain three subfractions (Fr. 3-1 to 3-3). Fr. 3-2 was further separated via semi-pHPLC (40% MeCN-H_2_O) to obtain compounds 1 (9.2 mg, t*_R_* 9.6 min) and 3 (11.9 mg, t*_R_* 13.1 min). Fr. 3-3 was also purified, using semi-pHPLC (65% MeOH-H_2_O), to yield compounds 2 (10.6 mg, t*_R_* 15.4 min) and 4 (18.1 mg, t*_R_* 11.5 min).

The EtOAc extract of the rice fermentation was fractionated via using the same above-mentioned method to obtain four fractions (Frs. 1–4). Fr. 3 was purified via CC over RP-C18, eluting with a MeOH-H_2_O gradient (from 2:8 to 1:0) to obtain four subfractions (Fr. 3-1 to 3-4). Fr. 3-2 was further separated via CC over silica gel, eluting with a petroleum ether–acetone gradient (10:1, 5:1, 2:1, and 1:1) to yield compounds 8 (4.3 mg), 9 (2.1 mg), and 10 (3.4 mg). Compound 5 (14.3 mg) was prepared from Fr. 3-3 using semi-pHPLC (45% MeOH-H_2_O), while compounds 6 (7.7 mg) and 7 (6.2 mg) were purified from Fr. 3-4 using Sephadex LH-20 (Acetone) and semi-pHPLC (55% MeOH-H_2_O).

*Trichomide D* (compound 1): Colorless crystal. Melting point 221–222 °C; [${\left[\alpha \right]}_{D}^{24}$ = −78.2, *c* 0.35, MeOH; ^1^H and ^13^C NMR data, see Table 1; HRESIMS *m*/*z* 680.3399 [M + Na]^+^ (calcd for NaC_31_H_52_N_5_O_8_Cl, 680.3397).

*Cyclonerodiol C* (compound 8): White powder. ${\left[\alpha \right]}_{D}^{24}$ = −28.3, *c* 0.86, CHCl_3_; ^1^H and ^13^C NMR data, see Table 2 and Table S1; HRESIMS *m*/*z* 321.2042 [M + Na]^+^ (calcd for NaC_17_H_30_O_4_, 321.2036).

*Cyclonerodiol D* (compound 9): White powder. [${\left[\alpha \right]}_{D}^{24}$ = −32.6, *c* 1.03, CHCl_3_; ^1^H and ^13^C NMR data, see Table 2; HRESIMS *m*/*z* 279.1930 [M + Na]^+^ (calcd for NaC_15_H_28_O_3_, 279.1931).

### 3.5. Crystal Structure Determination

A colorless single crystal of compound 1 was obtained by the slow evaporation of a methanol solution (containing trace water), thus, its crystal structure contained two molecules of H_2_O. H_2_O molecules can form intermolecular hydrogen bonds with the cyclodepsipeptide compound 1, which was helpful to the crystallization of the compound. All crystallographic data were collected at 150.01 K on a Bruker Smart-1000 CCD diffractometer (Bruker-AXS, Saarbrucken, Germany) equipped with graphite-monochromatic Cu-Kα radiation (λ = 1.54178 Å). The adsorption data were obtained using the program SADABS [39]. The structures were elucidated by direct methods, using the SHELXTL software package [40]. All non-hydrogen atoms were refined with anisotropic displacement parameters. The hydrogen atoms were located via geometrical calculations, and their positions and thermal parameters were fixed during structure refinement. The structures were refined using full-matrix least-squares techniques [41]. Crystallographic data of compound 1 was deposited in the Cambridge Crystallographic Data Centre as CCDC 1858313.

*Crystal data for compound* 1: C_31_H_52_N_5_O_8_Cl·2H_2_O, FW = 694.25, Monoclinic, space group P 1 21 1, unit cell dimensions *a* = 10.1814(3) Å, *b* = 11.3775(4) Å, *c* = 15.5997(5) Å, *α* = *β* = *γ* = 90°, V = 1806.82(10) Å^3^, *Z* = 2, *d*_calcd_ = 1.276 mg/m^3^, crystal dimensions 0.32 × 0.25 × 0.14 mm, *μ* = 1.436 mm^−1^, *F*(000) = 748. The 26927 measurements yielded 6124 independent reflections after equivalent data were averaged, and Lorentz and polarization corrections were applied. The final refinement yielded *R*_1_ = 0.0489 and *wR*_2_ = 0.1237[*I* > 2*σ*(*I*)]. The Flack parameter was 0.024 (13) in the final refinement for all 6124 reflections with 443 Friedel pairs. There were also crystallographic disorders of *δ*-CH_2_−Cl/*δ*’-CH_2_−Cl in the residue of *δ*-Cl-DHPA^1^, probably due to the flexibility of the *δ*-Cl-DHPA^1^ residue.

### 3.6. Cytotoxicity against Human Cancer Cell Lines

The in vitro cytotoxic effects of the novel compounds 1, 8, and 9 were evaluated on five human cancer cell lines using the MTT (3-(4,5-dimethylthiazol-2-yl)-2,5-diphenyltetrazolium bromide) method [32,33,34]. The human cancer cell lines were as follows: HL-60, human myeloid leukemia; A-549, lung cancer; MCF-7, breast cancer; SW-480, human colon cancer; and SMMC-7721, liver cancer. All cells were cultured in RPMI-1640 medium containing 10% fetal bovine serum (FBS), and kept in a humidified atmosphere containing 5% CO_2_ at 37 °C. The novel compounds and the cisplatin positive control were dissolved and diluted in DMSO to obtain sample solvents with a series of different concentrations. In brief, the cell suspensions (200 μL, 5 × 10^4^ cells/mL) were seeded into 96-well culture plates and kept at 37 °C for 12 h, then the sample solvents (20 μL) were added into each well and further cultured at 37 °C for 24 h. Subsequently, MTT (100 μg) was added into each well and incubated at 37 °C for 4 h. After removal of the 100 μL culture medium, the cells were lysed with 20% SDS-50% DMF (100 μL). The remaining lysates were subjected to measurements of the optical density at 595 nm with a 96-well microtiter plate reader. Reed and Muench’s method was used to calculate IC_50_ values [32].

### 3.7. Brine Shrimp Lethality and Nematicidal Activity

Brine shrimp (*Artemia salina*) toxicity was evaluated as previously reported [36,37]. The plant-parasitic nematode *H. avenae* was selected for nematicidal bioassay using 24-well plates. Second stage juveniles (J2s) of *H. avenae* were collected to prepare the nematode suspension based on the protocol reported previously [34]. The isolated compounds (1–10) and abamectin positive control were dissolved and diluted in DMSO to obtain sample solvents with a series of different concentrations. The sample solvents (5 μL) were added to separate wells with the nematode suspension (495 μL), containing about 100 J2s, while the same amount of DMSO (5 μL) was added for the negative control. The plates were maintained at 25 °C for 48 h, and then observed using a stereomicroscope to evaluate the nematode mortalities. Nematodes were defined to be dead if their bodies became straight and did not react to mechanical touches [38]. The experiment was repeated three times under the same conditions.

### 3.8. Antifungal Activity

The isolated compounds (1–10) were also evaluated for antifungal activity against three plant pathogenic fungi, *R. cerealis*, *V. mali*, and *F. oxysporum* f. sp. *vasinfectum*, using the broth microdilution method [34,42].

## 4. Conclusions

The chemical investigation of marine-derived fungus *T. roseum* resulted in isolation of trichomide cyclodepsipeptides (compounds 1–4) from the liquid PDB medium, and, based on the OSMAC culture strategy, isolation of destruxin cyclodepsipeptides (compounds 5–7) and cyclonerodiol sesquiterpenes (compounds 8–10) from solid rice medium. The absolute configuration of novel compound 1 was determined by single crystal X-ray diffraction analysis, while the configurations of compounds 8 and 9 were determined by NOESY experiments, comparisons of specific rotations, ECD calculation, and plausible biosynthetic pathways. The novel compound 1 exhibited significant cytotoxic activities against human cancer cell lines MCF-7, SW480, and HL-60, with IC_50_ values of 0.079, 0.107, and 0.149 μM, respectively. In addition, it also showed significant brine shrimp lethality with an LD_50_ value of 0.48 μM, and moderate nematicidal activity against *H. avenae* with an LC_50_ value of 94.9 μg/mL. Its cytotoxic, brine shrimp lethality and nematicidal activities suggest potential applications in the areas of medicine and agriculture. This is also the first time the cytotoxic and nematicidal potential of trichomide cyclodepsipeptides has been reported.

## Figures and Tables

**Table 1:** ^1^H (500 MHz) and ^13^C (125 MHz) NMR data of compound 1 (CD_3_OD, *δ*: ppm).

No.	*δ*_C_ (type)	*δ*_H_ (mult., *J* in Hz)	No.	*δ*_C_ (type)	*δ*_H_ (mult., *J* in Hz)
	*δ*-Cl-DHPA^1^			*N*-Me-Ile^4^	
CO	170.4, C		CO	172.1, C	
*α*	70.7, CH	5.21, t (7.0)	*α*	56.9, CH	5.09, d (11.0)
*β*	34.9, CH_2_	2.18, m	*β*	33.4, CH	2.06, m
*γ*	67.2, CH	3.82, m	*β*-Me	14.8, CH_3_	0.90, overlapped
*δ*	48.3, CH_2_	3.61, d (5.0)	*γ*	25.3, CH_2_	1.50, m; 1.07, m
	*β*-Me-Pro^2^		*δ*	10.0, CH_3_	0.94, overlapped
CO	172.4, C		*N*-Me	30.1, CH_3_	3.21, s
*α*	67.5, CH	4.08, m		*N*-Me-Ala^5^	
*β*	37.5, CH	2.55, m	CO	170.8, C	
*γ*	30.6, CH_2_	2.10, m; 1.77, br s	*α*	55.4, CH	5.32, q (6.5)
*δ*	45.4, CH_2_	4.06, m; 3.95, m	*β*	14.3, CH_3_	1.32, overlapped
*β*-Me	17.9, CH_3_	1.16, d (6.8)	*N*-Me	27.5, CH_3_	2.71, s
	Ile^3^			*β*-Ala^6^	
CO	173.6, C		CO	173.6, C	
*α*	53.4, CH	4.76, overlapped	*α*	33.8, CH_2_	2.69, m; 2.58, m
*β*	37.0, CH	1.94, m	*β*	33.4, CH_2_	3.80, m; 3.15, m
*β*-Me	14.3, CH_3_	0.86, overlapped	*NH*		8.56, br. s
*γ*	24.6, CH_2_	1.49, m;1.33, overlapped			
*δ*	9.7, CH_3_	0.87, overlapped			
*NH*		7.03, d (7.8)			

**Table 2:** ^1^H (500 MHz) and ^13^C (125 MHz) NMR data of compounds 8 and 9 (*δ*: ppm) ^a^.

Compound 8			Compound 9		
No.	*δ*_C_ (type)	*δ*_H_ (mult., *J* in Hz)	No.	*δ*_C_ (type)	*δ*_H_ (mult., *J* in Hz)
1	14.0, CH_3_	1.05 (6.6)	1	14.5, CH_3_	1.05 (6.8)
2	44.1, CH	1.57, m	2	44.3, CH	1.61, m
3	80.7, C		3	81.4, C	
4	40.0, CH_2_	1.63, m; 1.55, m	4	40.4, CH_2_	1.68, m; 1.53, m
5	23.8, CH_2_	1.85, m; 1.60, m	5	24.4, CH_2_	1.86, m; 1.61, m
6	54.1, CH	1.87, m	6	54.2, CH	1.86, m
7	74.1, C		7	74.5, C	
8	40.0, CH_2_	1.52, t (8.4)	8	43.4, CH_2_	2.20, m
9	22.0, CH_2_	2.13, m	9	122.1, CH	5.70, overlapped
10	129.6, CH	5.47, t (7.0)	10	142.3, CH	5.70, overlapped
11	129.9, C		11	70.8, C	
12	69.9, CH_2_	4.45, s	12	29.9, CH_3_	1.33, s
13	24.7, CH_3_	1.26, s	13	26.1, CH_3_	1.25, s
14	23.2, CH_3_	1.17, s	14	25.2, CH_3_	1.13, s
15	12.5, CH_3_	1.67, s	15	29.9, CH_3_	1.33, s
COCH_3_	19.4, CH_3_	2.08, s			
C=O	171.5, C				

^a^ 8 and 9 were determined using CDCl_3_ and CD_3_OD, respectively.

**Table 3:** Cytotoxicity of the new compound 1 against five human cancer cell lines (IC_50_: μM).

Compound	MCF-7	SW480	HL-60	A-549	SMMC-7721
1	0.079 ± 0.004	0.107 ± 0.015	0.149 ± 0.007	>40	>40
Cisplatin	19.44 ± 1.57	20.80 ± 1.04	3.72 ± 0.09	16.97 ± 0.69	12.35 ± 0.52

**Table 4:** Brine shrimp lethality (LD_50_, μM) and nematicidal activity (LC_50_, μg/mL) of compounds 1–10.

	1	2	3	4	5	6	7	8	9	10	Positive Control
brine shrimp lethality	0.48	0.74	>50	3.22	2.47	2.81	>50	n.a.	n.a.	n.a.	8.4 ^a^
nematicidal activity	94.9	143.6	>500	221.8	207.7	293.4	>500	n.a.	n.a.	n.a.	23.1 ^b^

n.a.: no activity. ^a,b^ colchicine for brine shrimp lethality and abamectin for the nematicidal bioassay.

## Supplementary Materials

The following are available online at http://www.mdpi.com/1660-3397/16/12/519/s1, Table S1: ^1^H (500 MHz) and ^13^C (125 MHz) NMR data of compound 8 (DMSO-*d*6); Table S2: Antifungal activities of compounds 1–10 (MIC, μg/mL); Figure S1: HPLC analyses of crude extracts of the liquid PDB and solid rice media; Figures S2, S8, and S19: HRESIMS spectrum of compound 1, 8, and 9, respectively; Figures S3–S7, S9–S18, and S20–S24: annotated 1D NMR and selected 2D NMR spectra of 1, 8, and 9; Figure S25: Comparison of ECD spectrum for (2*S*, 3*R*, 6*R*, 7*R*, and 9*E*)-9 with the experimental one of 9 in MeOH.
