# Supplementary material for: Cyclodepsipeptides and Sesquiterpenes from Marine-Derived Fungus Trichothecium roseum and Their Biological Functions
Source: Mar Drugs. 2018 Dec 19;16(12):519. doi: 10.3390/md16120519 (PMC6316070; doi:10.3390/md16120519)
Supplement: Supplementary file 1 [file marinedrugs-16-00519-s001.pdf]

Supplementary Materials: Cyclodepsipeptides and Sesquiterpenes from Marine-Derived Fungus *Trichothecium roseum* and Their Biological Functions

Yuan-Ming Zhou, Guang-Lin Ju, Lin Xiao, Xiang-Fei Zhang, and Feng-Yu Du

Contents

Table S1. ^{1}H (500 MHz) and ^{13}C (125 MHz) NMR data of compound 8 (DMSO-*d6*).	S2
Table S2. Antifungal activities of compounds 1–10 (MIC, μg/mL).	S2
Figure S1. HPLC analyses of crude extracts of the liquid PDB and solid rice media.	S3
Figure S2. HRESIMS spectrum of compound 1.	S4
Figure S3. ^{1}H NMR (500 MHz, CD_3OD) spectrum of compound 1.	S4
Figure S4. DEPT spectrum of compound 1.	S5
Figure S5. ^{1}H-^{1}H COSY spectrum of compound 1.	S5
Figure S6. HSQC spectrum of compound 1.	S6
Figure S7. HMBC spectrum of compound 1.	S6
Figure S8. HRESIMS spectrum of compound 8.	S7
Figure S9. ^{1}H NMR (500 MHz, CD_3OD) spectrum of compound 8.	S7
Figure S10. ^{13}C NMR spectrum of compound 8 (CD_3OD).	S8
Figure S11. ^{1}H-^{1}H COSY spectrum of compound 8 (CD_3OD).	S8
Figure S12. HSQC spectrum of compound 8 (CD_3OD).	S9
Figure S13. HMBC spectrum of compound 8 (CD_3OD).	S9
Figure S14. ^{1}H NMR (500 MHz, DMSO-*d6*) spectrum of compound 8.	S10
Figure S15. ^{13}C NMR spectrum of compound 8 (DMSO-*d6*).	S10
Figure S16. HSQC spectrum of compound 8 (DMSO-*d6*).	S11
Figure S17. HMBC spectrum of compound 8 (DMSO-*d6*).	S11
Figure S18. NOESY spectrum of compound 8 (DMSO-*d6*).	S12
Figure S19. HRESIMS spectrum of compound 9.	S12
Figure S20. ^{1}H NMR (500 MHz, $CDCl_3$) spectrum of compound 9.	S13
Figure S21. DEPT spectrum of compound 9.	S13
Figure S22. ^{1}H-^{1}H COSY spectrum of compound 9.	S14
Figure S23. HSQC spectrum of compound 9.	S14
Figure S24. HMBC spectrum of compound 9.	S15
Figure S25. Comparison of ECD spectrum for (2*S*, 3*R*, 6*R*, 7*R*, and 9*E*)-9 with the experimental one of 9 in MeOH.	S15

Table S1. ^{1}H (500 MHz) and ^{13}C (125 MHz) NMR data of compound 8 (DMSO-*d6*).

Position	δ_C (type)	δ_H (mult., *J* in Hz)
1	15.5, CH_3	0.92 (6.7)
2	44.3, CH	1.47, m
3	79.6, C	
4	40.9, CH_2	1.53, m; 1.37, m
5	24.5, CH_2	1.69, m; 1.50, m
6	54.3, CH	1.68, m
7	73.2, C	
8	40.6, CH_2	1.36, m
9	22.5, CH_2	1.98, m
10	130.1, CH	5.45, t (7.4)
11	129.8, C	
12	69.8, CH_2	4.40, s
13	26.7, CH_3	1.12, s
14	25.1, CH_3	1.02, s
15	14.2, CH_3	1.61, s
$COCH_3$	21.2, CH_3	2.03, s
C=O	170.7, C	
3–OH		3.81, s
7–OH		3.89, s

Table S2. Antifungal activities of compounds 1–10 (MIC, μg/mL).

Compounds	*R. cerealis*	*V. mali*
Trichomide cyclodepsipeptides		
1	128	256
2	—	—
3	—	—
4	256	128
Destruxin cyclodepsipeptides		
5	—	—
6	—	—
7	—	—
Cyclonerodiol sesquiterpenes		
8	128	128
9	128	64
10	—	—
Tebuconazole[a]	8	16

[a] Positive control: tebuconazole;

Figure S1. HPLC analyses of crude extracts of the liquid PDB and solid rice media.

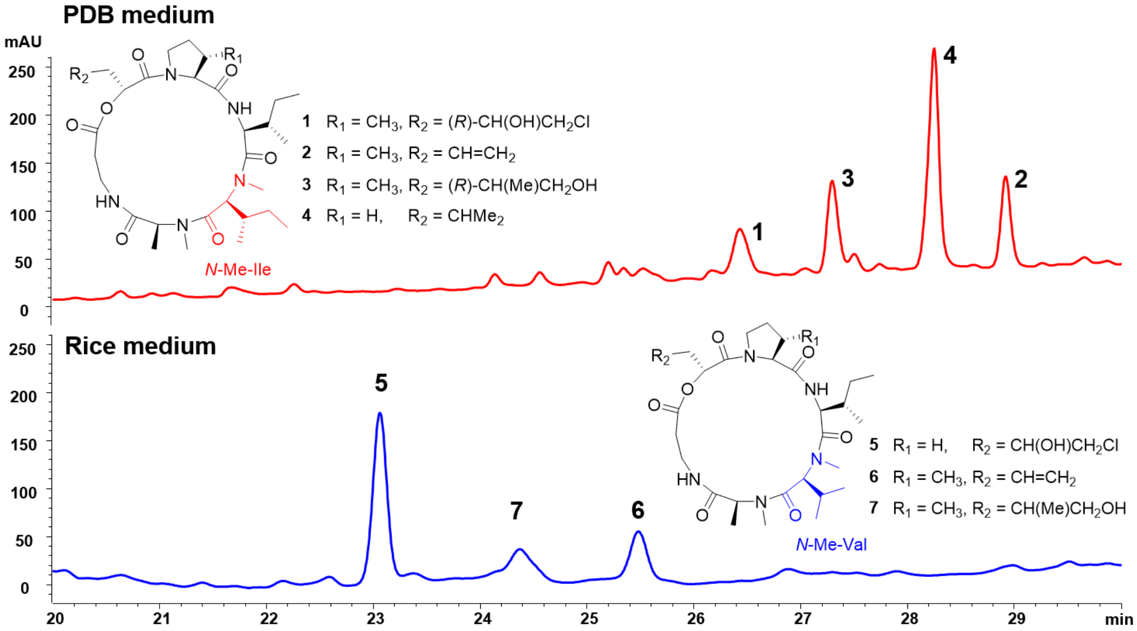

Chromatographic conditions: mobile phases: solvents A (MeOH) and B (H_2O); eluting method: 10% solvent A in B for the first 0 to 2 min, then increase to 100% A at 30 min, followed by 3 min with 100% A; flow rate: 1.0 mL/min; UV detection: 225 nm.

Results: The HPLC analysis of the crude extract of the liquid PDB medium showed four significant peaks (compounds 1–4) at the time range of 20–30 min, while the solid rice medium further exhibited three new peaks (compounds 5–7). Due to the very weak UV absorptions of compounds 8–10 at 225 nm, they did not show obvious peaks in the HPLC analysis of the rice medium. However, significant colored spots from compounds 8–10 could be observed in the TLC analyses of the subfractions derived from the crude extract of the rice medium.

Figure S2. HRESIMS spectrum of compound 1.

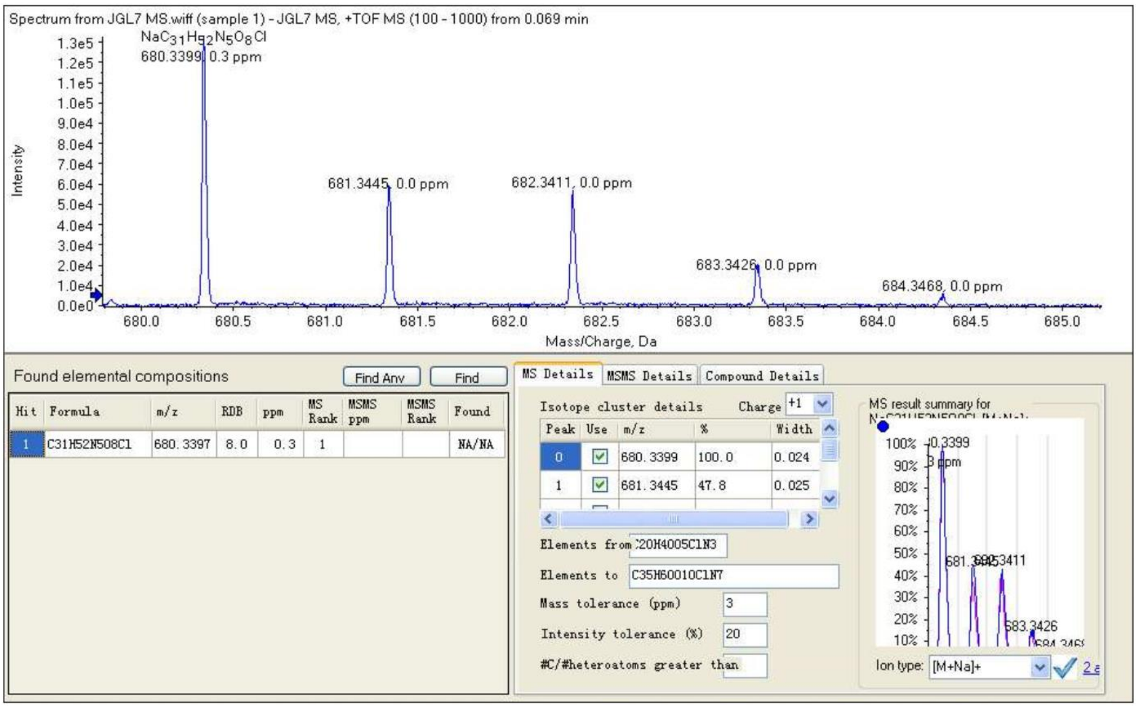

Figure S3. ^{1}H NMR (500 MHz, CD$_3$OD) spectrum of compound 1.

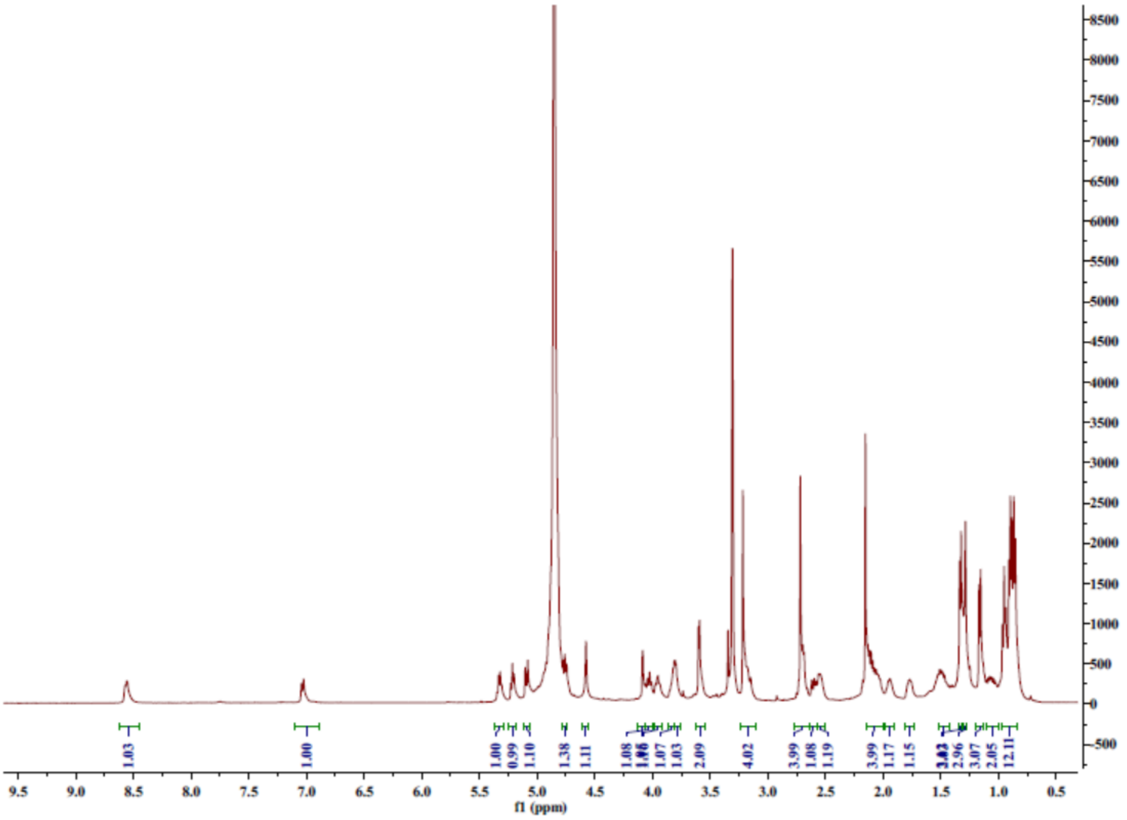

Figure S4. DEPT spectrum of compound 1.

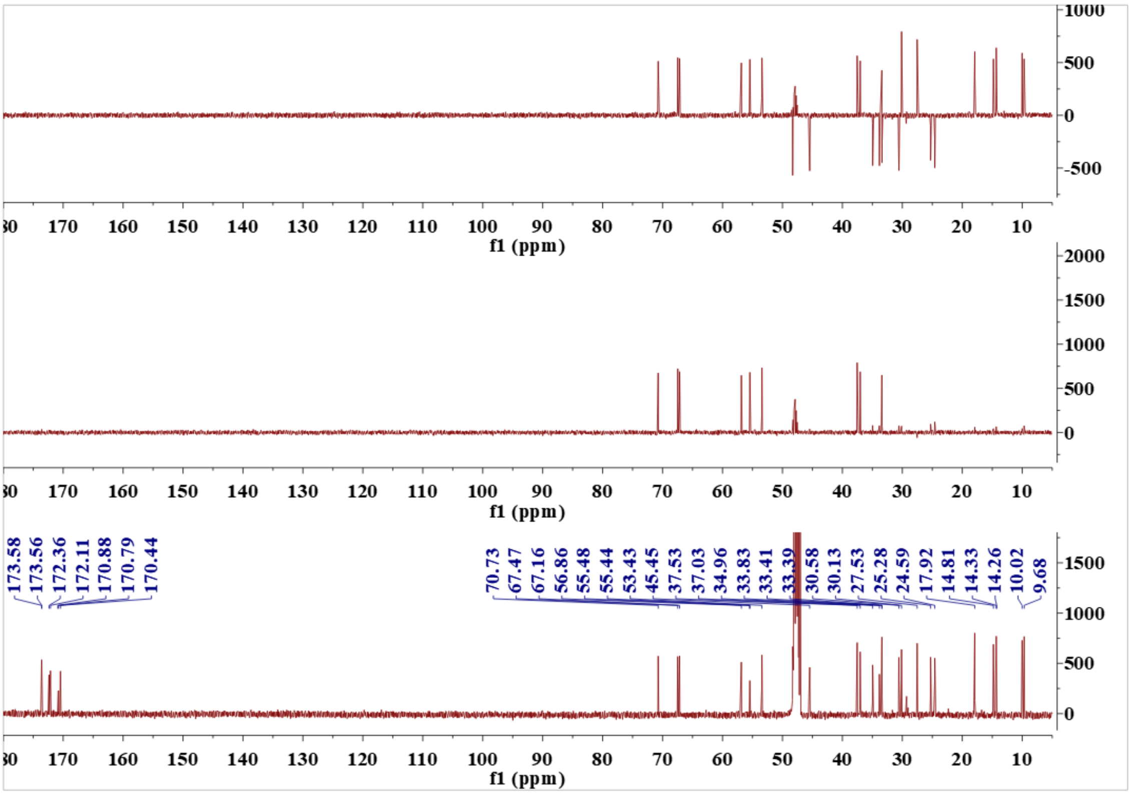

Figure S5. ^{1}H–^{1}H COSY spectrum of compound 1.

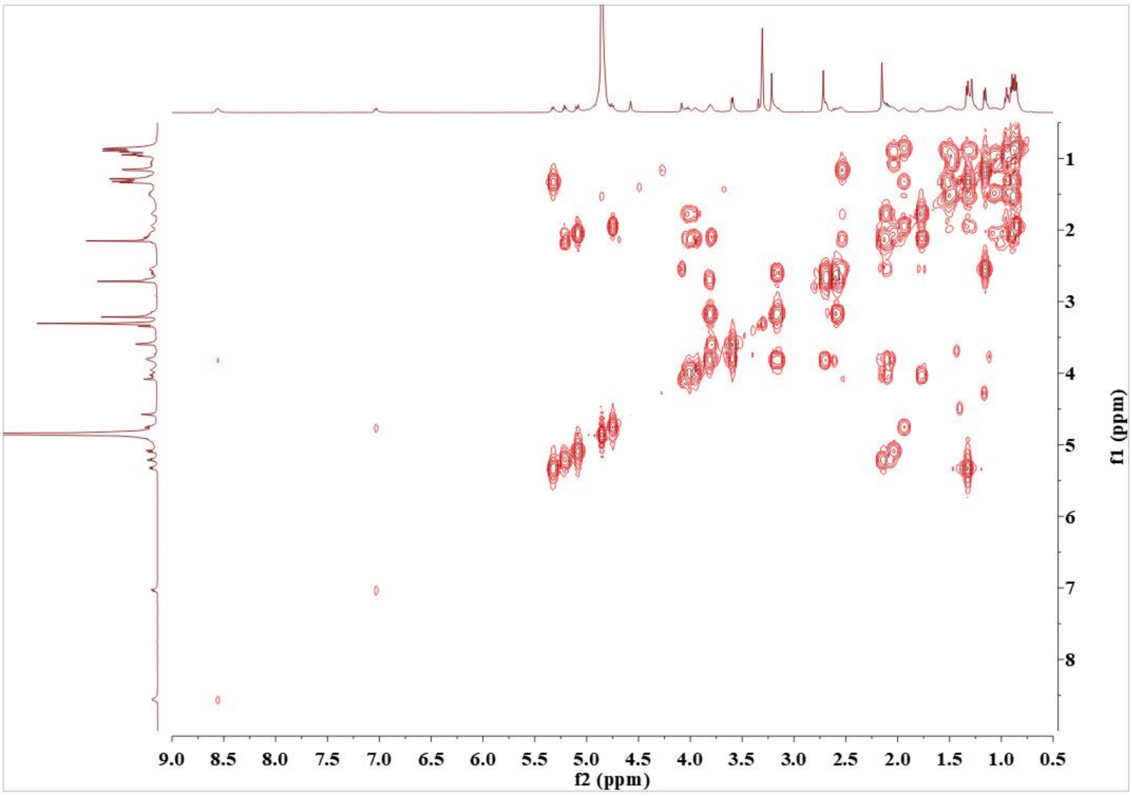

Figure S6. HSQC spectrum of compound 1.

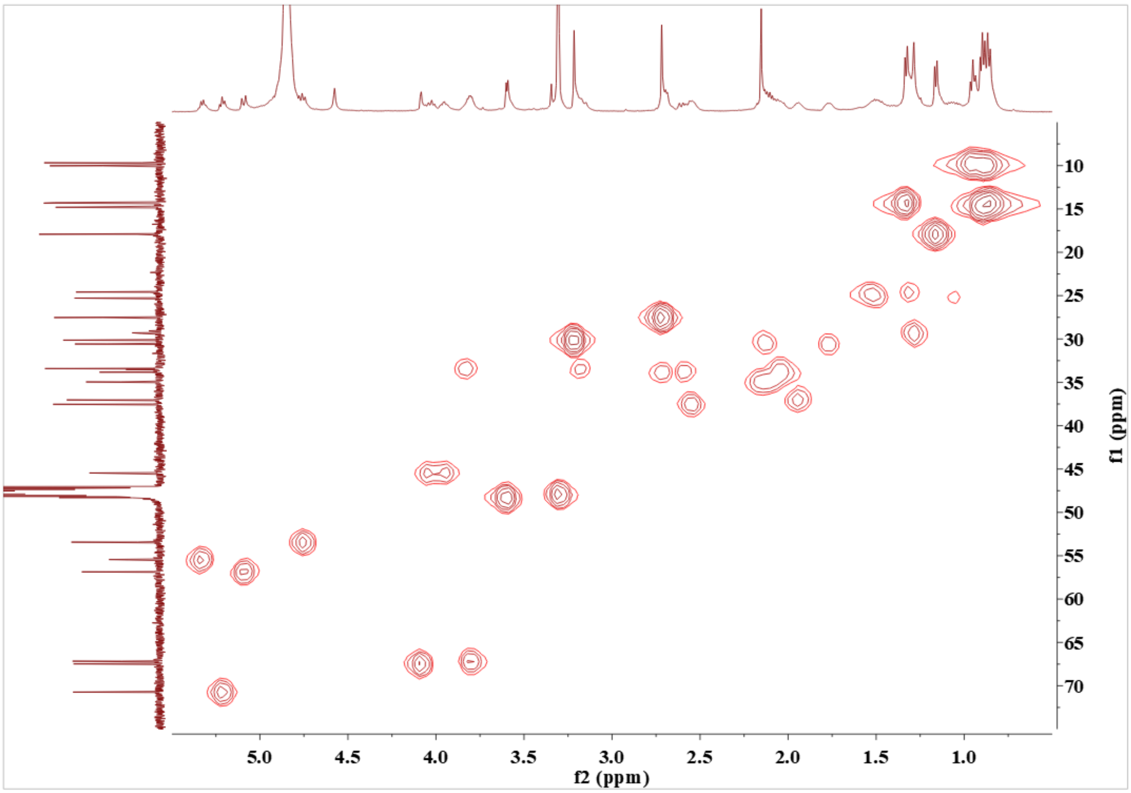

Figure S7. HMBC spectrum of compound 1.

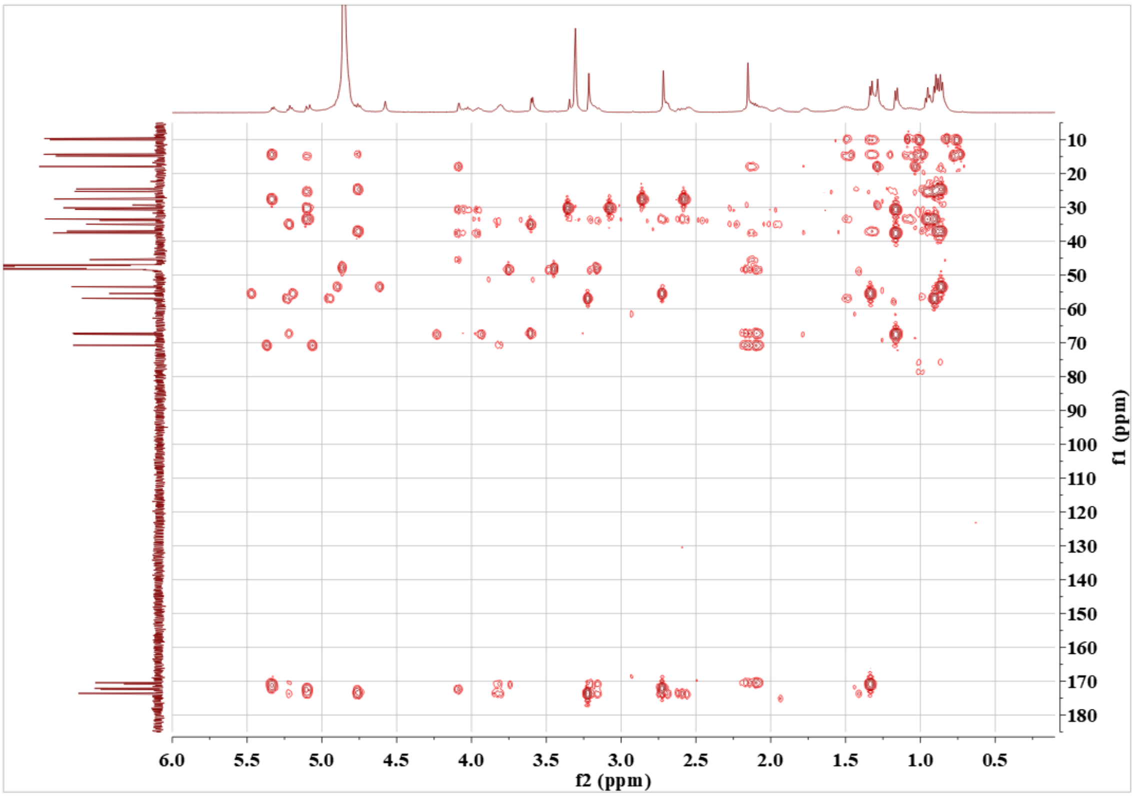

Figure S8. HRESIMS spectrum of compound 8.

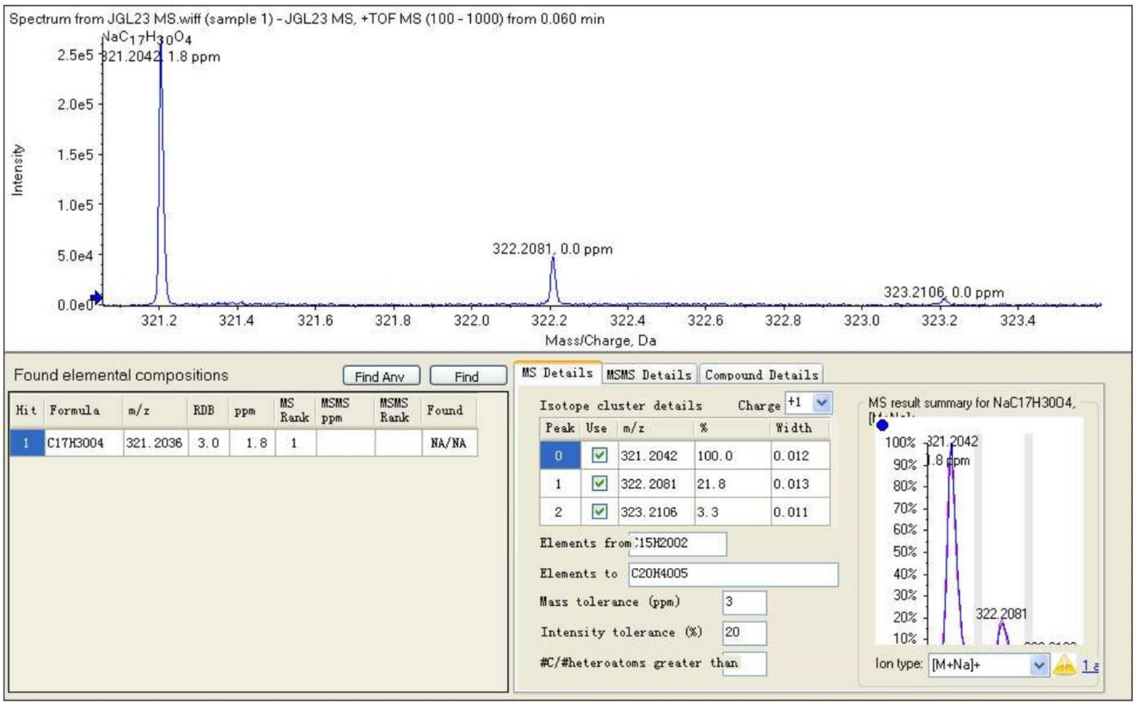

Figure S9. ^{1}H NMR (500 MHz, CD_3OD) spectrum of compound 8.

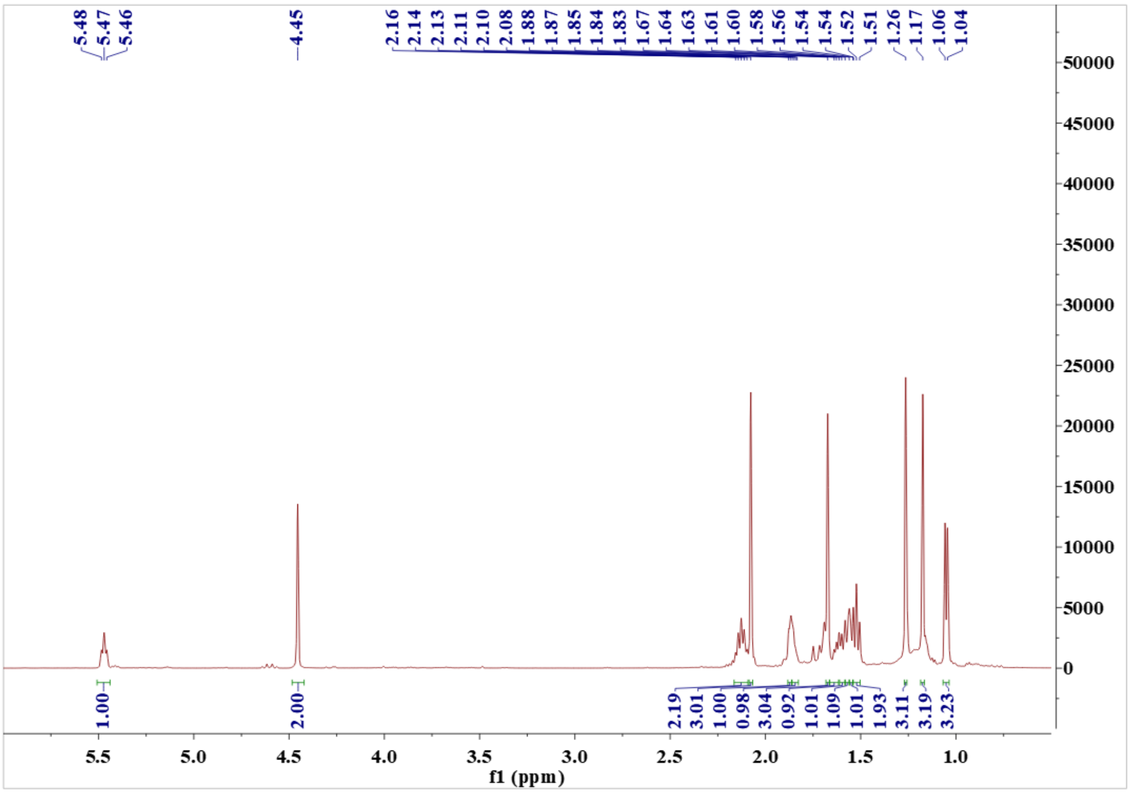

Figure S10. ^{13}C NMR spectrum of compound 8 (CD_3OD).

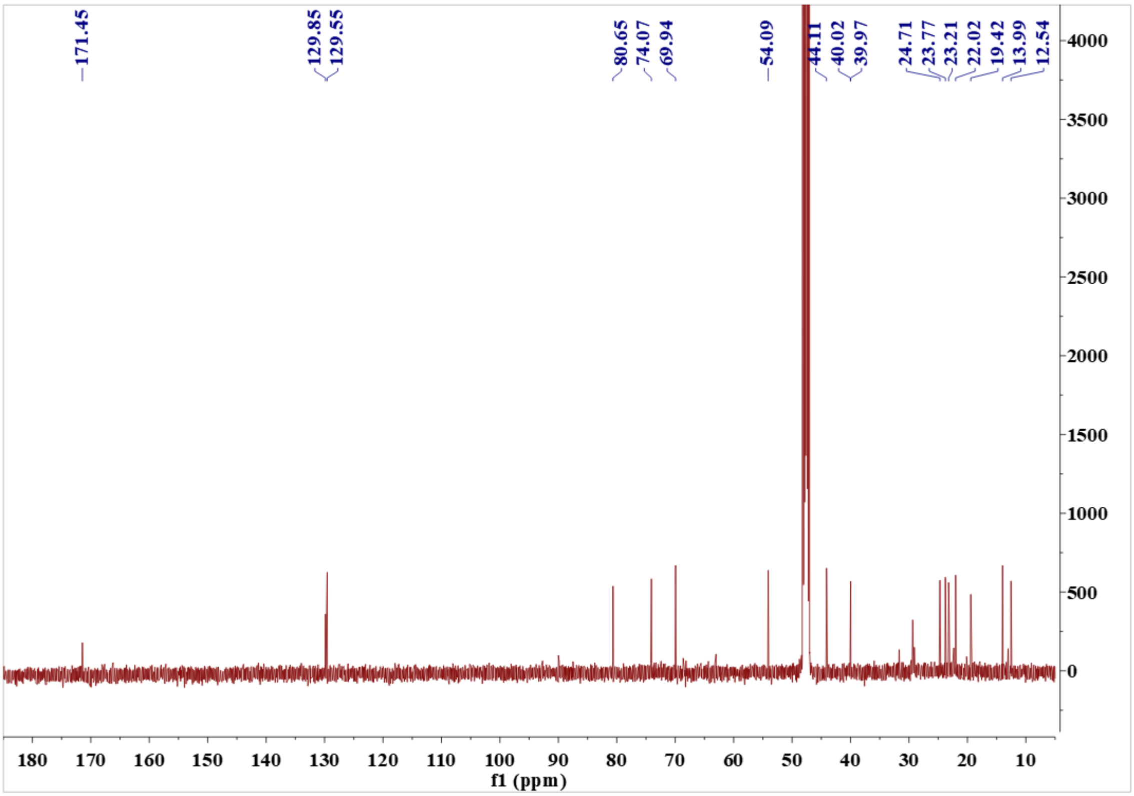

Figure S11. ^{1}H–^{1}H COSY spectrum of compound 8 (CD_3OD).

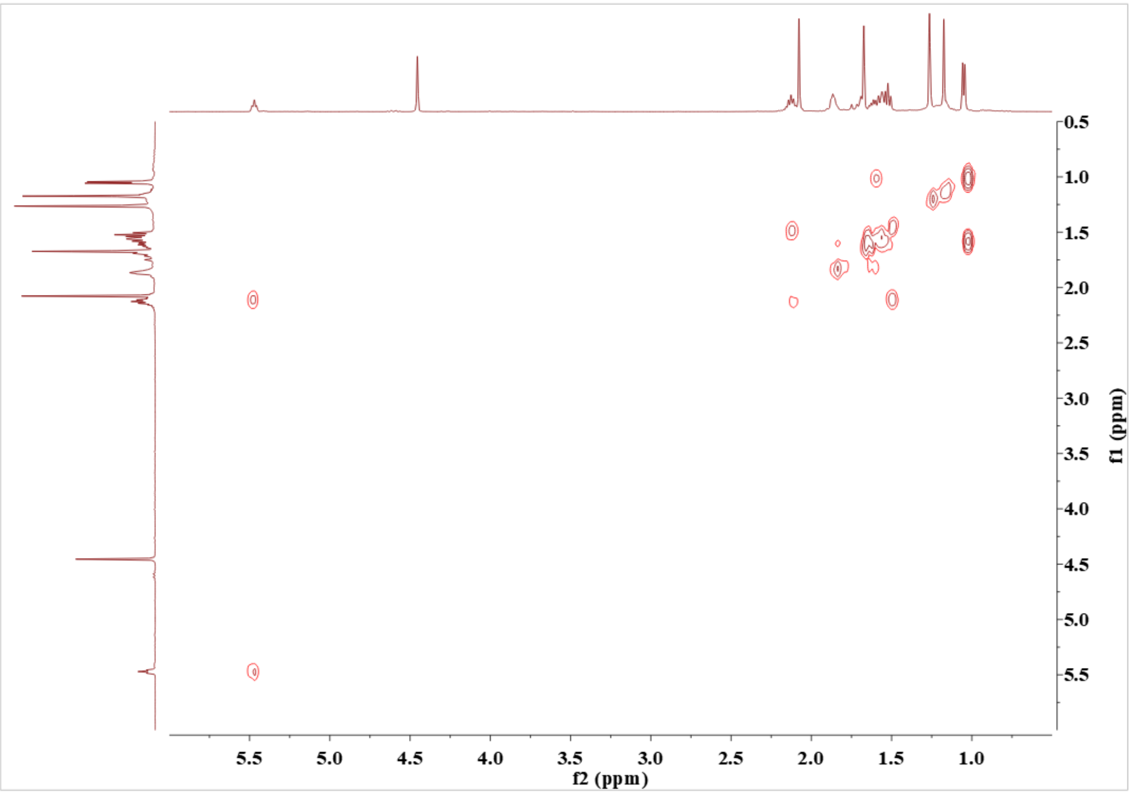

Figure S12. HSQC spectrum of compound 8 (CD_3OD).

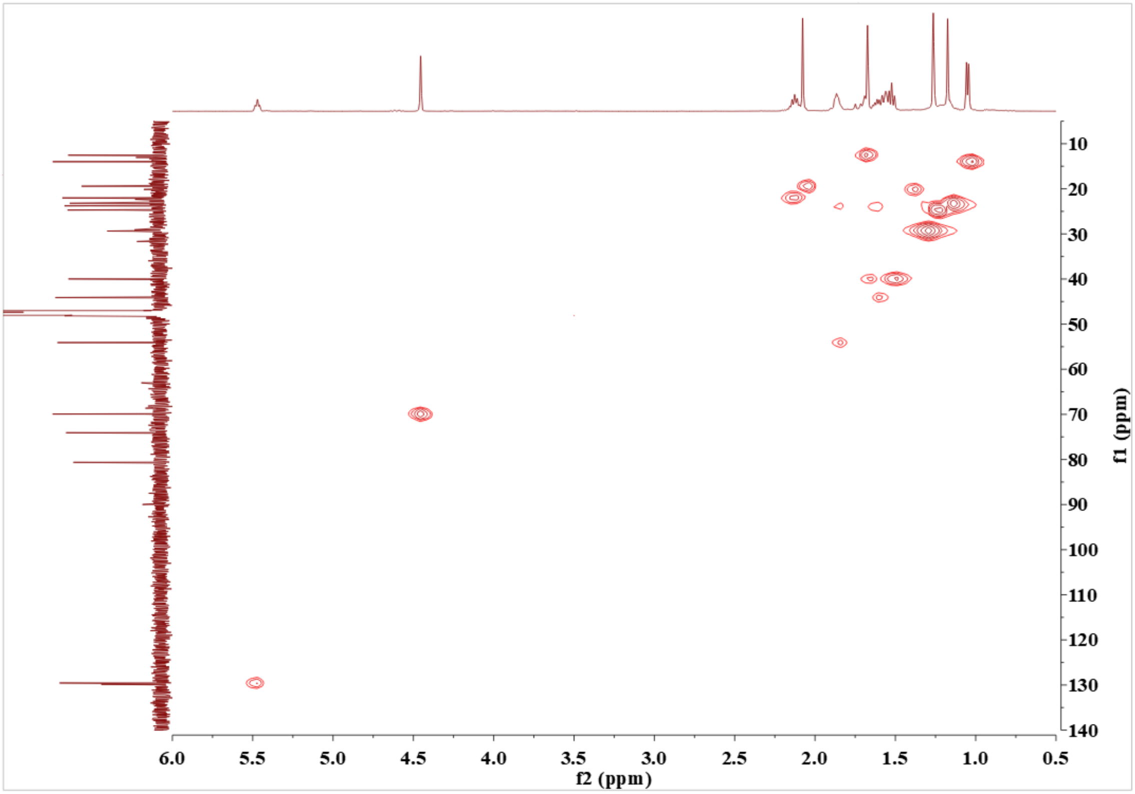

Figure S13. HMBC spectrum of compound 8 (CD_3OD).

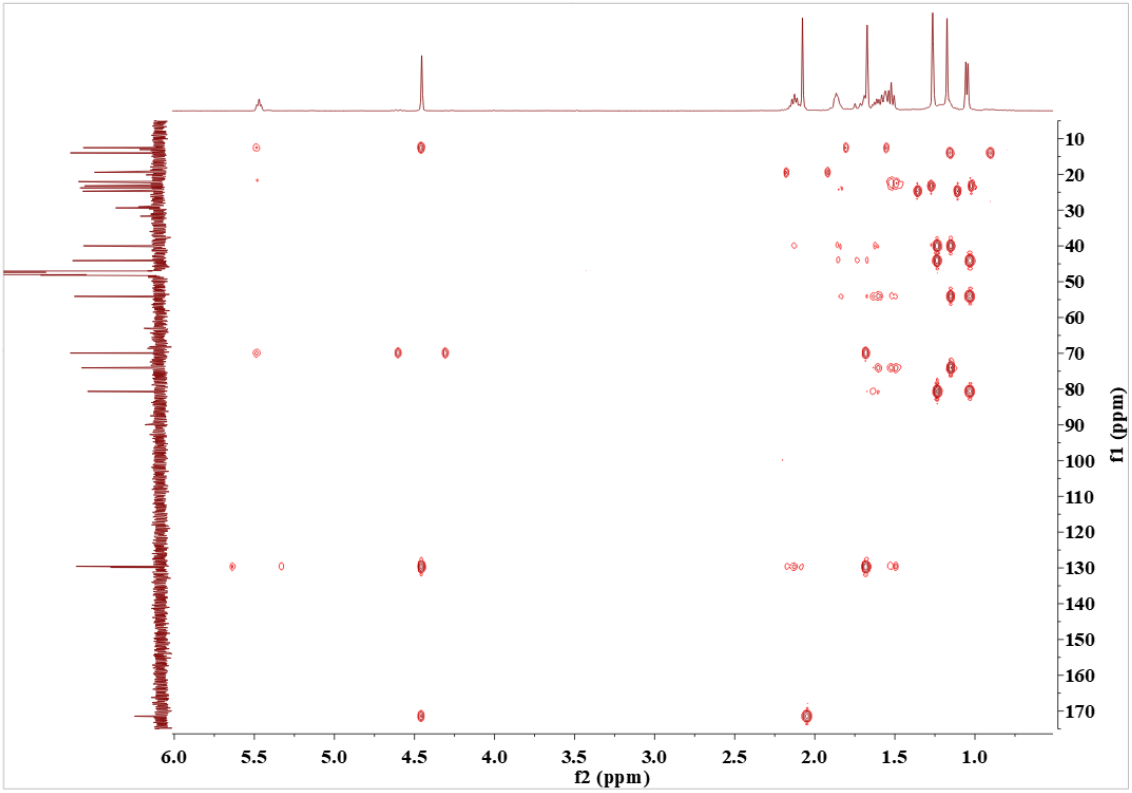

Figure S14. ^{1}H NMR (500 MHz, DMSO-*d6*) spectrum of compound 8.

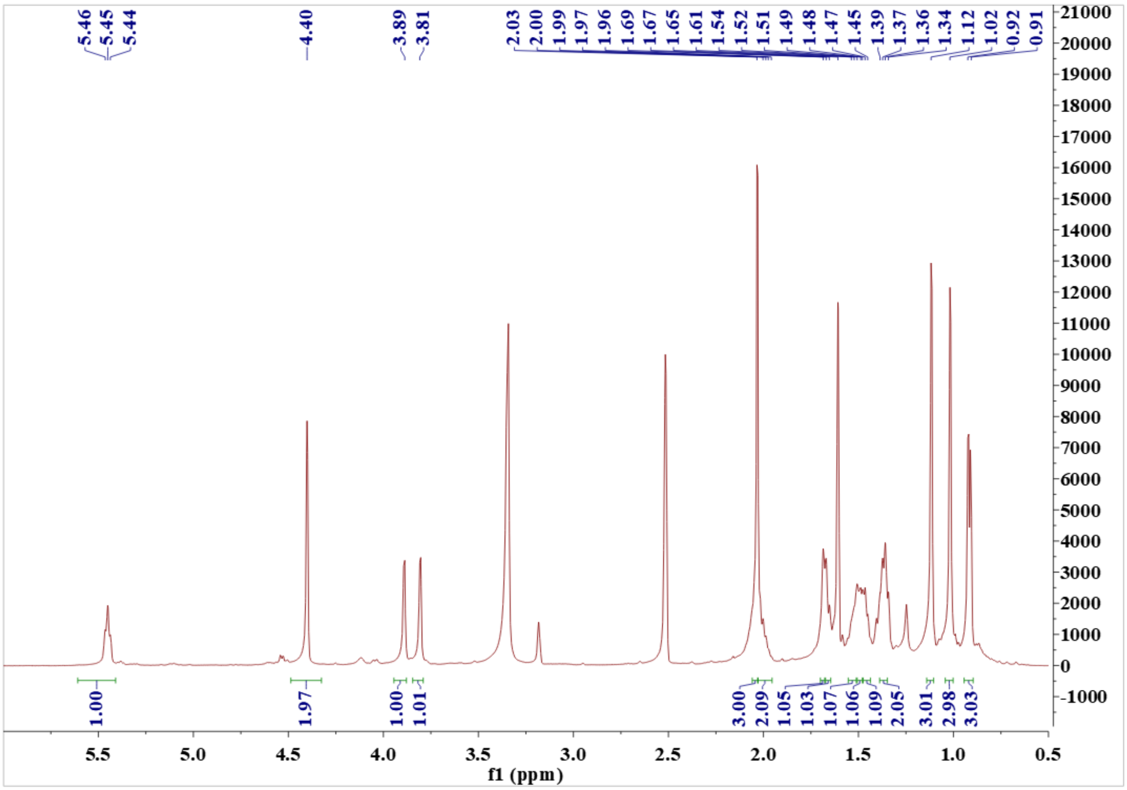

Figure S15. ^{13}C NMR spectrum of compound 8 (DMSO-*d6*).

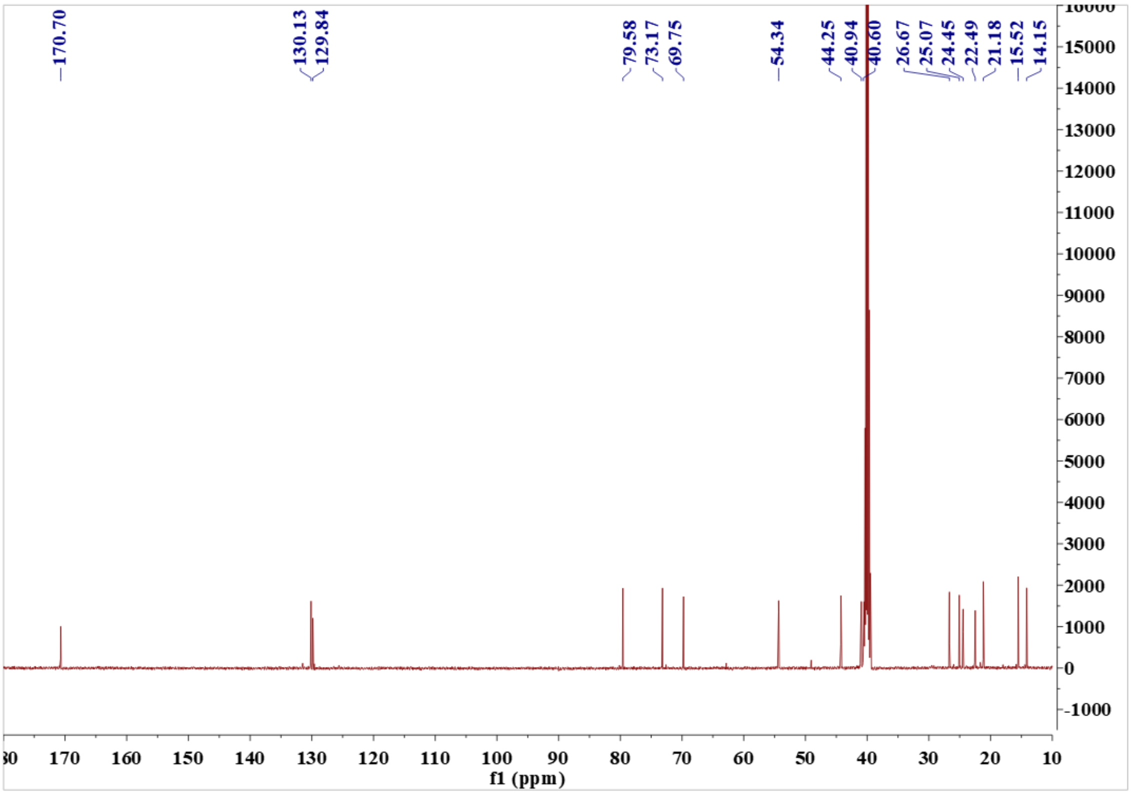

Figure S16. HSQC spectrum of compound 8 (DMSO-*d6*).

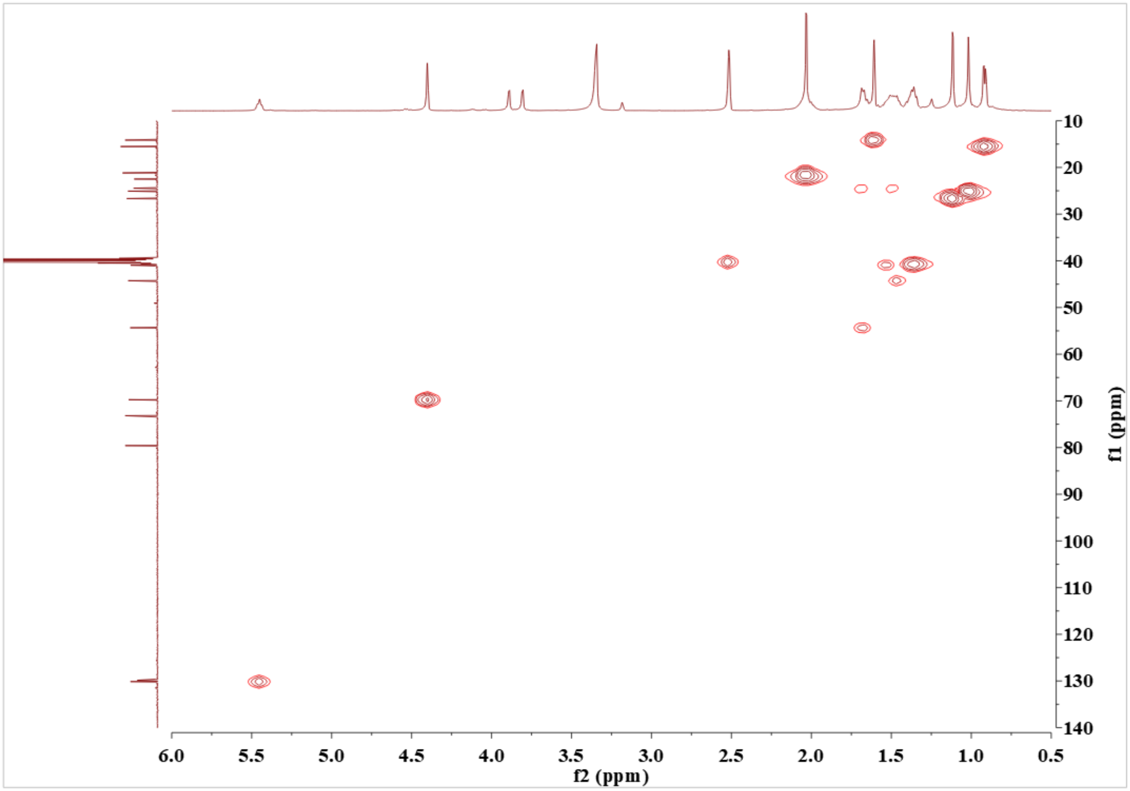

Figure S17. HMBC spectrum of compound 8 (DMSO-*d6*).

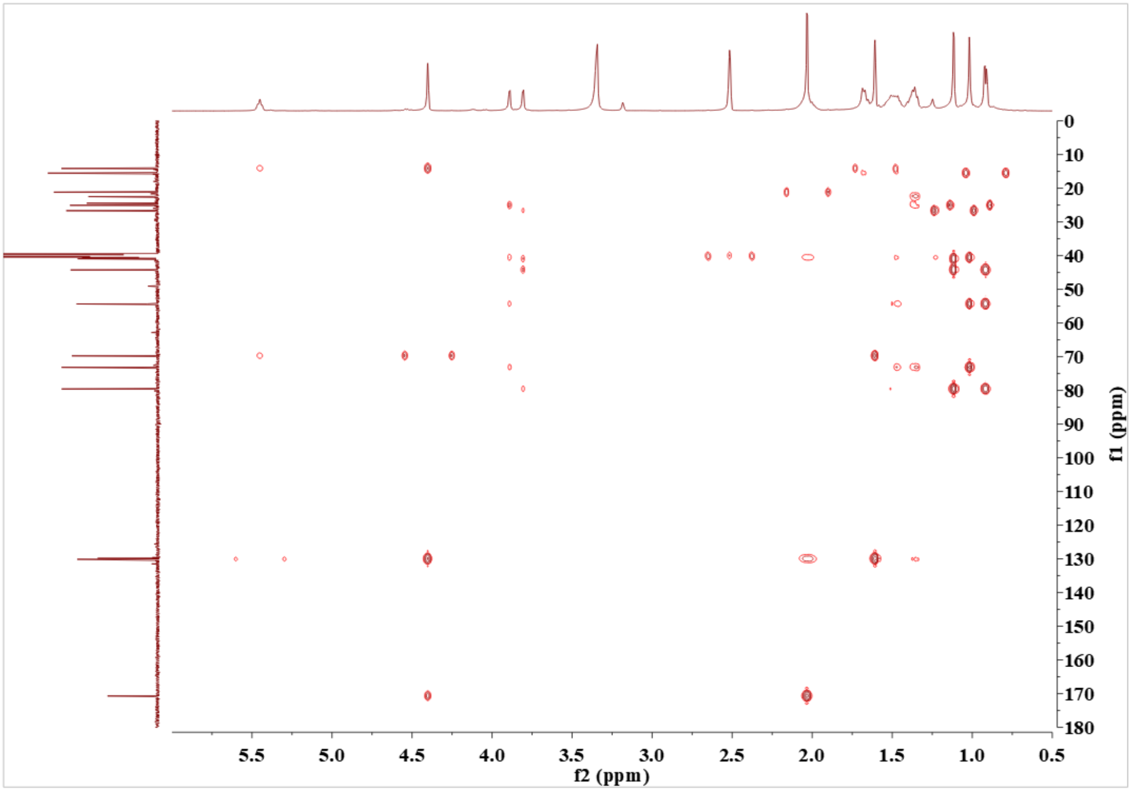

Figure S18. NOESY spectrum of compound 8 (DMSO-*d6*).

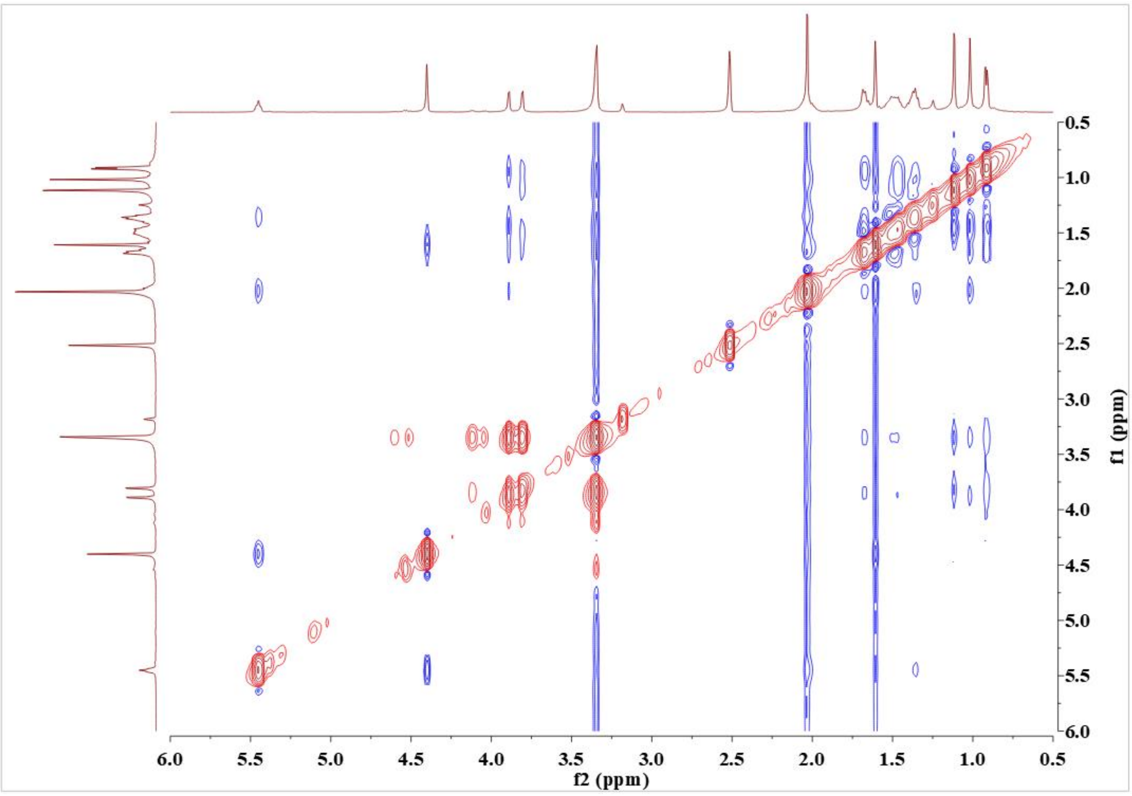

Figure S19. HRESIMS spectrum of compound 9.

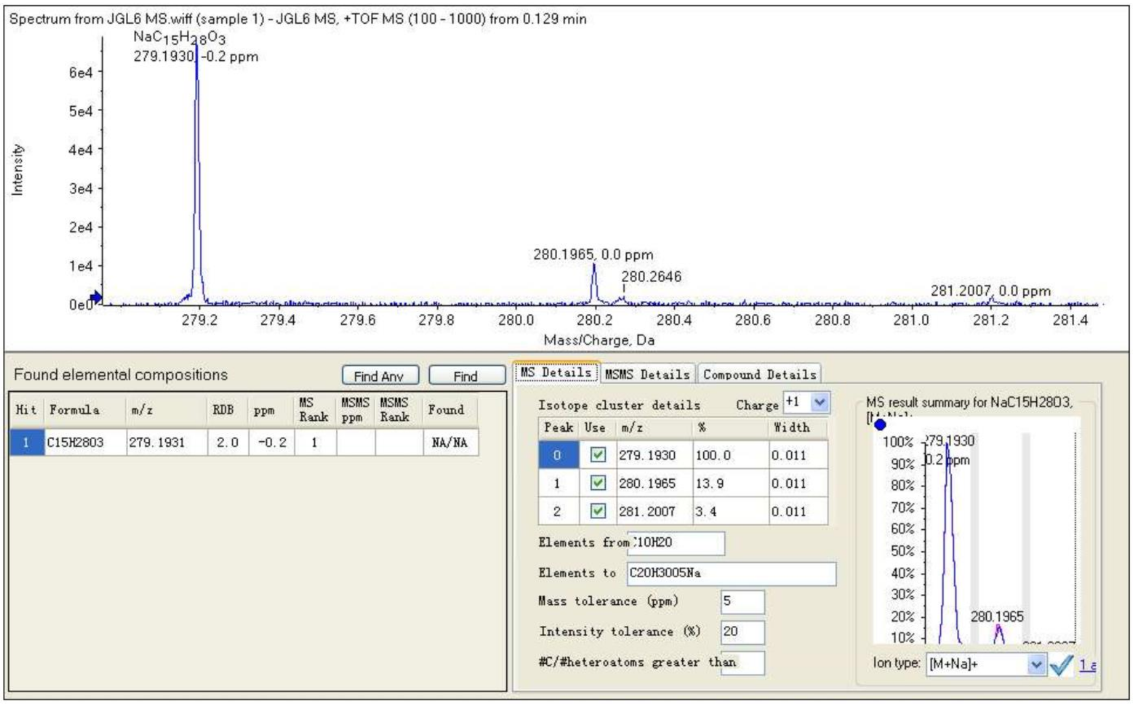

Figure S20. ^1H NMR (500 MHz, $CDCl_3$) spectrum of compound 9.

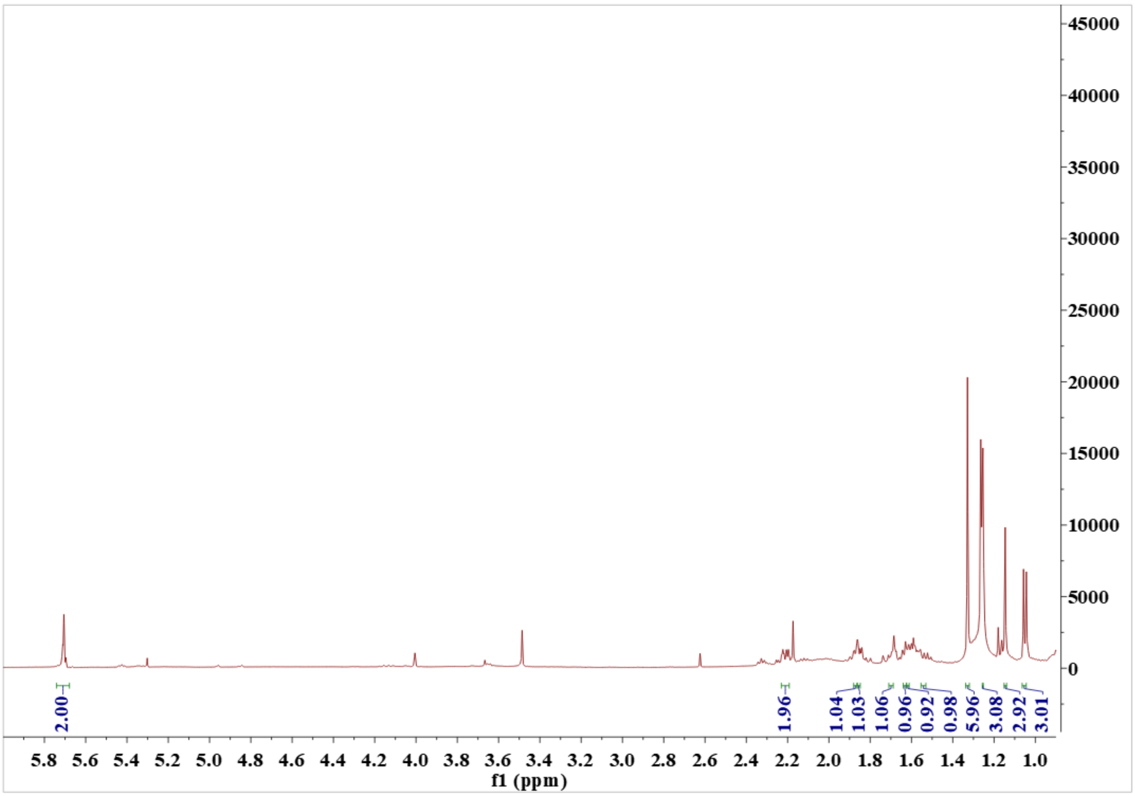

Figure S21. DEPT spectrum of compound 9.

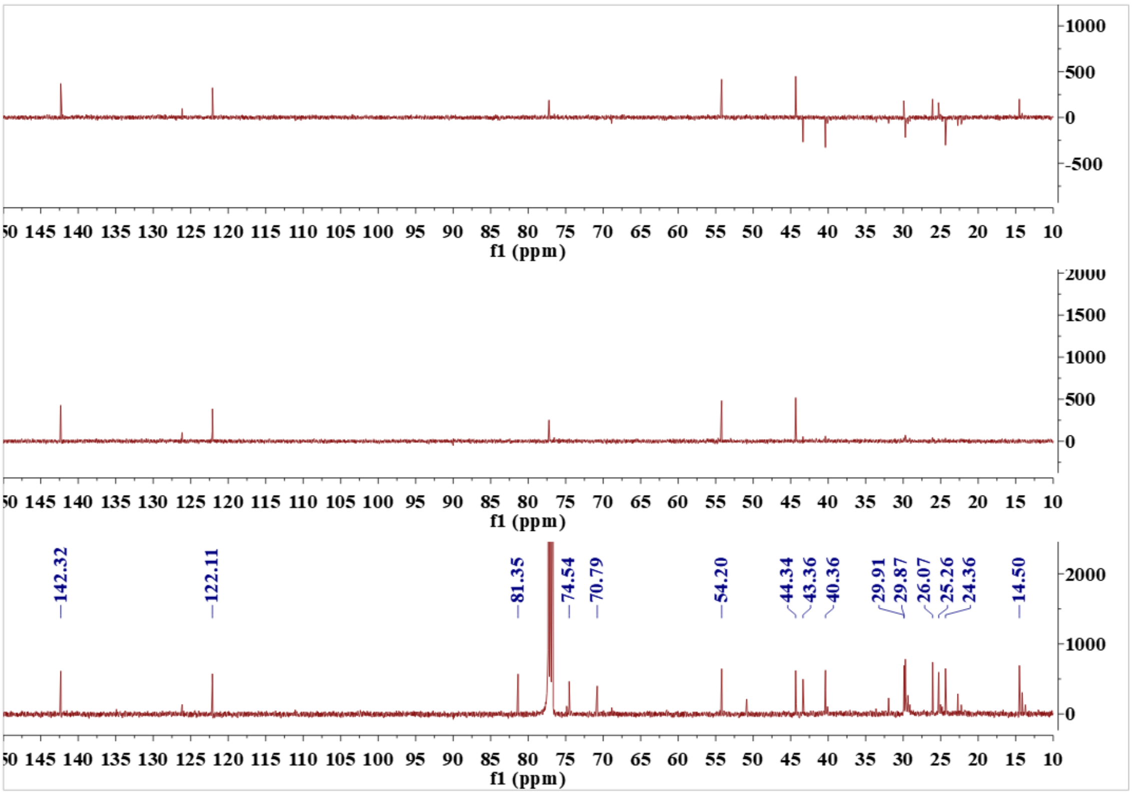

Figure S22. ^{1}H–^{1}H COSY spectrum of compound 9.

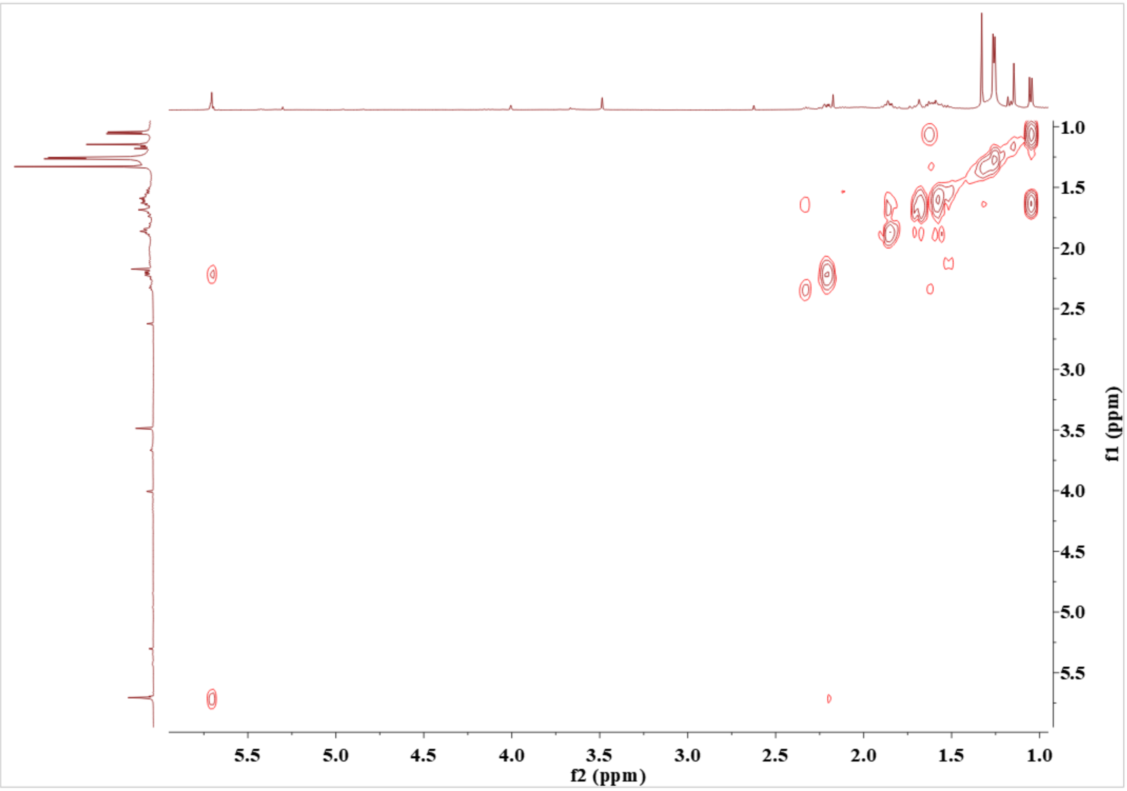

Figure S23. HSQC spectrum of compound 9.

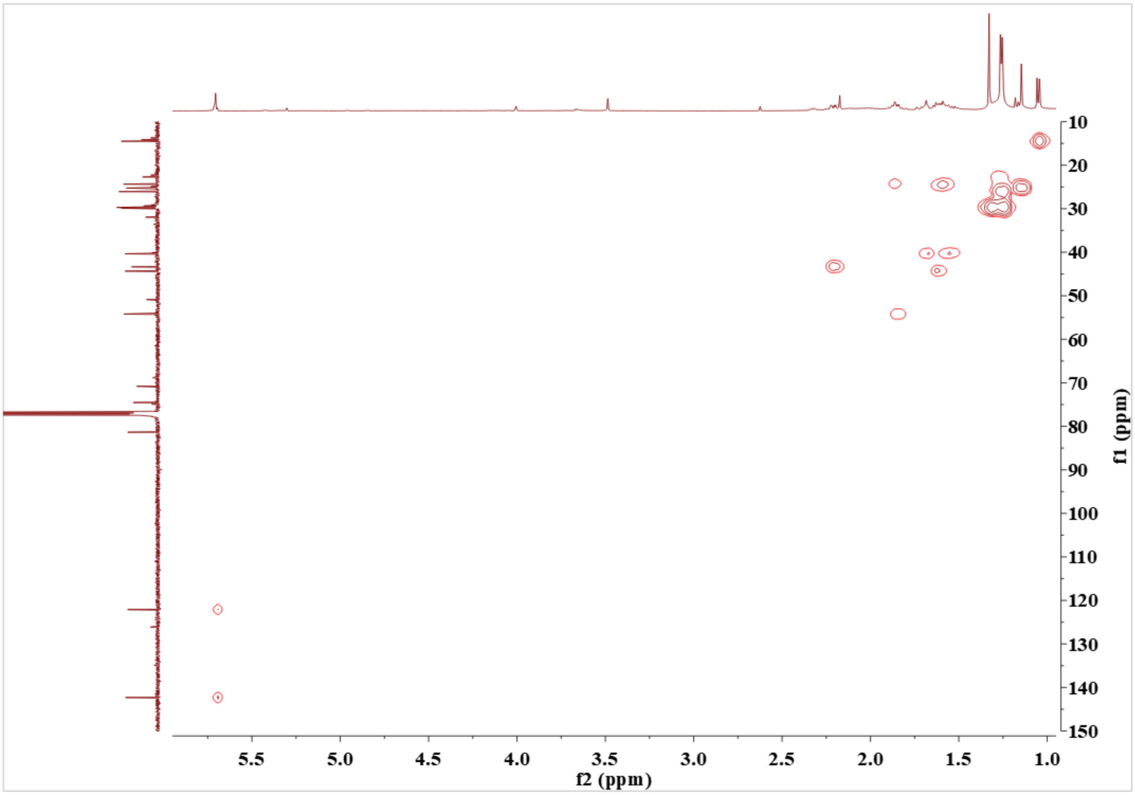

Figure S24. HMBC spectrum of compound 9.

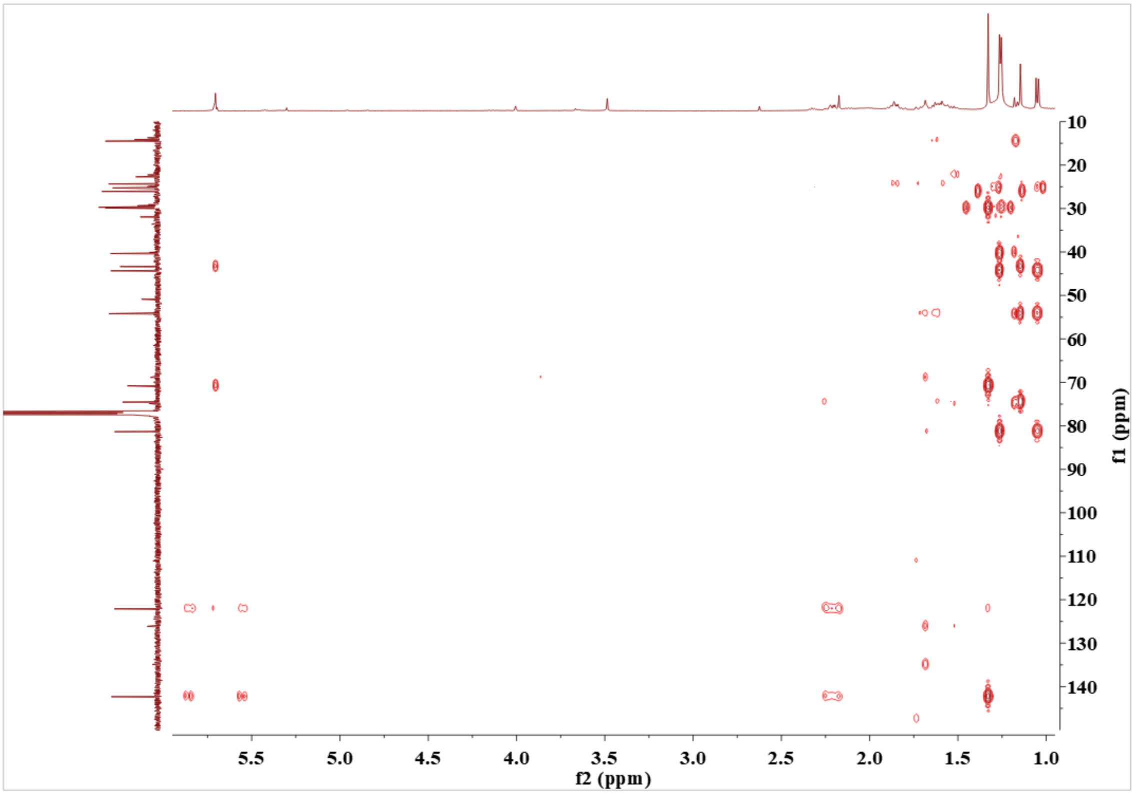

Figure S25. Comparison of ECD spectrum for (2*S*, 3*R*, 6*R*, 7*R*, and 9*E*)-9 with the experimental one of 9 in MeOH.

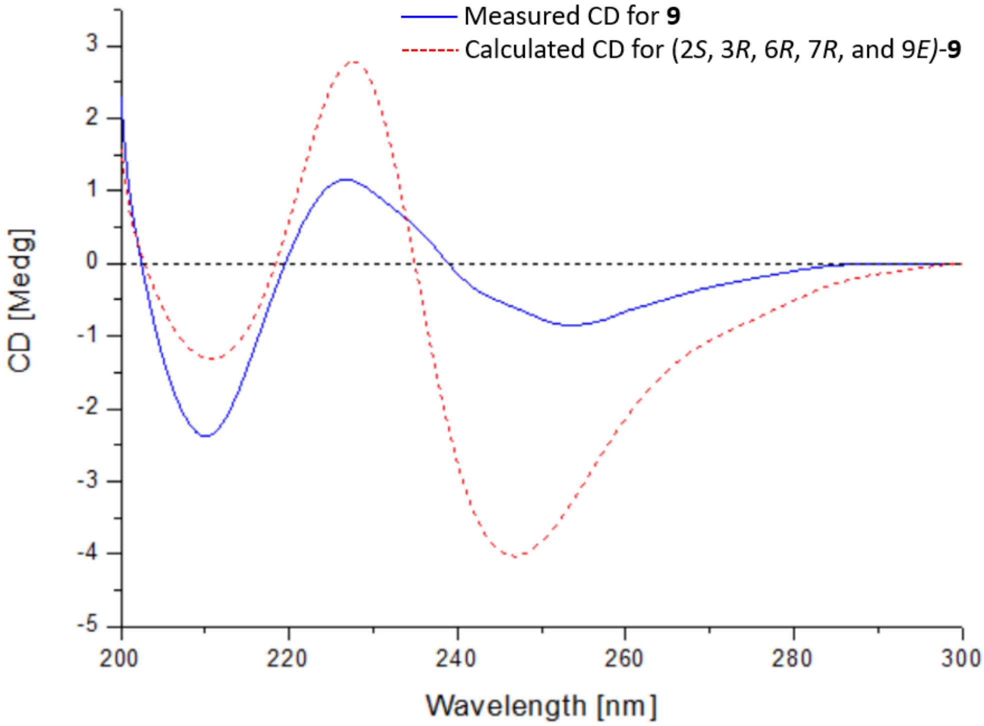

Method: Monte Carlo conformational searches were carried out using the Spartan's 10 software using Merck Molecular Force Field (MMFF). The conformers with a Boltzmann population of over 5% were

chosen for ECD calculations, and then the conformers were initially optimized at B3LYP/6-31+g (d, p) level in MeOH, using the CPCM polarizable conductor calculation model. The theoretical calculation of ECD was conducted in MeOH using time-dependent density functional theory (TD-DFT) at the B3LYP/6-311+g (d, p) level for all conformers of compound 9. Rotatory strengths for a total of 50 excited states were calculated. ECD spectra were generated using the program SpecDis 1.6 (University of Würzburg, Würzburg, Germany) and GraphPad Prism 5 (2365 Northside Dr., Suite 560, San Diego, CA 92108) from dipole-length rotational strengths, by applying Gaussian band shapes with sigma = 0.3 eV.
